# Assessment of risk, landscape epidemiology and management strategies to combat alveolar echinococcosis in the rural communities of Hunza, Pakistan

**DOI:** 10.3389/fpubh.2022.1015475

**Published:** 2022-11-21

**Authors:** Naila Jamill, Haroon Ahmed, Muhammad Sohail Afzal, Sami Simsek, Abid Ali, Muhammad Arshad, Chenghang Yu, Jianping Cao

**Affiliations:** ^1^Department of Biosciences, COMSATS University Islamabad, Islamabad, Pakistan; ^2^Department of Life Sciences, School of Science, University of Management and Technology, Lahore, Pakistan; ^3^Department of Parasitology, Faculty of Veterinary Medicine, Firat University, Elazig, Turkey; ^4^Department of Zoology, Abdul Wali Khan University Mardan, Mardan, Pakistan; ^5^National Institute of Parasitic Diseases, Chinese Center for Disease Control and Prevention (Chinese Center for Tropical Diseases Research), Shanghai, China; ^6^Key Laboratory of Parasite and Vector Biology, National Health Commission of the People's Republic of China, Shanghai, China; ^7^WHO Collaborating Center for Tropical Diseases, Shanghai, China; ^8^The School of Global Health, Chinese Center for Tropical Diseases Research, Shanghai Jiao Tong University School of Medicine, Shanghai, China

**Keywords:** alveolar echinococcosis, awareness, risk factors, disease management, Hunza, Pakistan

## Abstract

**Background:**

Human alveolar echinococcosis (AE) is a neglected zoonotic disease. Prevalence of AE in humans is reported in Pakistan as a result of poor economic and sanitary conditions, close proximity to wildlife and limited knowledge of AE. Studies on the prevalence and transmission of AE have been limited, especially for rural Pakistan. The study objectives were to identify knowledge, attitudes and practices relating to AE, to determine awareness of the disease, and to identify knowledge about possible risk factors of infections involving the landscape epidemiological attributes of rural villages in Hunza, one of the districts of Gilgit-Baltistan, a region of Pakistan that borders China.

**Methods:**

A community-based cross-sectional study was conducted among the general population of Hunza to establish the level of awareness, knowledge, attitudes, practices, landscape epidemiology, and disease management and control relating to AE in rural areas of Hunza. Data were collected by questionnaire.

**Results:**

A total of 387 questionnaires was received. Statistical analysis showed that the population's knowledge about the disease was poor. The attitudes and practices of the participants indicated that their risk of infection was low. Knowledge of landscape epidemiology of the disease was poor but knowledge about AE disease management was good. The attitudes of residents toward disease treatment and control strategies were positive, although the overall knowledge of participants about prevention of infection was poor.

**Conclusion:**

Knowledge of AE is poor among the residents of Hunza, Pakistan. Our study demands continued and strengthened awareness of the changes to lifestyle and practices associated with AE, not only in the study locality but throughout other areas of Pakistan.

## Background

Echinococcosis is a zoonotic parasite diseases caused by tapeworm species of the genus *Echinococcus* spp. There are four types of echinococcosis: (1) infection with a species complex centered on *Echinococcus granulosus sensu lato* that causes cystic echinococcosis (CE), often known as hydatid disease or hydatidosis; (2) alveolar echinococcosis (AE), induced by *Echinococcus multilocularis*; (3) polycystic neotropical echinococcosis, caused by *E. vogeli*; and (4) unicystic echinococcosis, caused by *E. oligarthrus*. CE and AE are the two most common forms with medical and public health implications in humans. Foxes, coyotes and dogs are host to the adult of *E. multilocularis*, whereas small wild rodents host the metacestodes (larvae). Infected dogs and foxes are the main source of infection in humans (1). *Echinococcus* has two mammalian hosts in its life cycle. The adult tapeworm lives in the small intestine of a carnivore (the definitive host) and generates oncospheres in the form of eggs. The carnivore's digestive tract releases either cestode segments (gravid proglottids) holding eggs or free eggs into the environment. In a larval stage, the metacestode develops in internal organs after an intermediate host takes eggs orally. The metacestode typically produces a large number of protoscoleces, each of which has the potential to develop into an adult cestode once ingested by a suitable final host. Humans are accidental hosts who do not usually play a role in the development cycle of the parasite. However, infection in humans results in serious morbidity and mortality if left untreated, along with substantial social and economic consequences (2).

AE is characterized by an asymptomatic incubation period of 5–15 years and the slow development of a primary tumor-like lesion, which is usually located in the liver, although it can extend to other organs. Because the cysts grow slowly, infection with AE may not cause symptoms for several years. As the cysts grow, they may cause pain or discomfort in the upper abdomen, weakness, weight loss, general malaise and signs of hepatic failure (3). Humans can acquire *E. multilocularis* eggs by contact with infected definitive hosts or consuming food or water contaminated with eggs (4). The incidence of the infection and the dynamics of transmission are further affected by the hosts' intrinsic vulnerability, the nature of their interactions, host population densities, seasonal fluctuations, host age, food diversity and other factors (5).

Till 1980s, only four countries in central Europe were known to have *E. multilocularis* endemic areas: Austria, France, Germany and Switzerland (6). During 1999–2000, parasite has a much wider geographic range, including at least 12 European countries: Austria, Belgium, the Czech Republic, Denmark, France, Germany, Liechtenstein, Luxembourg, Poland, the Slovak Republic, the Netherlands and Switzerland (7, 8). The recent report of *E. multilocularis* in eastern Poland and the Slovak Republic supports the concept that the endemic zones in central and eastern Europe, which were previously thought to be distinct, are now linked. According to a communication from animal health authorities in Troms and Finnmark, Norway, *E. multilocularis* metacestodes were reported in rats on Spitsbergen Island, which is part of the Norwegian Svalbard Island group in the Barents Sea, in 1999 (9). Between 1934 and 1983, 157 human cases of AE were diagnosed, resulting in an average of 3.1 new cases per year in Turkey (10).

It has been reported that 22.9% of red foxes (*Vulpes vulpes*) and 16% of jackals (*Canis aureus*) were infected with adult stages of *E. multilocularis* in Ardabile province, northern Iran. In addition, a total of 37 human AE cases were diagnosed in various hospitals in Iran over 3.5 years, the majority of which were in the Ardabile province (11).

In China, the incidence of AE was 257 cases in Ningxia, 88 cases in Xinjiang, 71 cases in Gansu, 49 cases in Sichuan, 37 cases in Qinghai, 1 case in Heilongjiang Tibet provinces in 1992 (12). In recent years, over 350 AE cases have been detected in the Gansu region alone. Between 1991 and 1997, an ultrasonography mass screening survey with serological confirmation in south Gansu demonstrated a group prevalence of human AE of about 4% (135/3331). Given the size of the population in this rural location, this equates to a local group prevalence of around 200 cases per 100,000. However, because AE has a focused distribution, group or local prevalence may not be indicative of prevalence in wider regions (13).

Because it is a zoonotic infectious disease, the success of AE prevention and control programs depends on the cooperation of people living in areas where the disease is common. For this reason, knowing the knowledge, attitudes and practices of the society about the disease is one of the important determinants of social participation in the implementation of prevention and control programs. Thus, the objective of the current study was to assess knowledge, attitudes and practices (KAPs) regarding AE, to determine awareness of AE in various villages in Hunza province of Pakistan, which borders neighboring areas of China.

## Methods

### Study area

The study focused on rural areas of Gilgit-Baltistan, which is located in the northern part of Pakistan. It is a mountainous region and covers an area of 72,971 km^2^ (14). Gilgit-Baltistan is divided into three main regions: Gilgit, Baltistan and Diamer. The Gilgit region has 14 districts, one of which is Hunza. This study was performed specifically in Hunza and included both the central and upper regions of the district. Hunza covers an area of 7,900 km^2^ and has a total population of 70,000 (15). Hunza is bounded north and east by the Kashgar area of China's Xinjiang Uyghur Autonomous Region. We selected 25 villages for our study, of which 14 are located in upper Hunza and 11 in central Hunza.

### Survey duration

Duration of the study was from August 2021 to December 2021. During this period, the various villages were visited to gather data on AE and identify AE risk factors.

### Calculation of sample size

The total population of the Hunza valley is about 46,665, of whom 46.92% are males and 53.08% are females. In terms of age distribution, 25% are <12 years, 57% are between 12 and 60 years and 18% are >60 years (15). A recommended sample size of 382, calculated at a confidence level of 95%, was determined using Raosoft online sample size calculator (16). No survey had been conducted before on AE; thus we did not have any preliminary data on the public health impact of AE in Gilgit-Baltistan.

### Study design

We used a community-based cross-sectional design for this study to determine awareness, prevalence and risk factors of AE disease in various rural areas of Hunza, Pakistan. A descriptive questionnaire was designed in such a way that students, professionals and villagers could complete it. Both qualitative and quantitative data were collected to establish the level of awareness and knowledge of AE disease among the population and their practices relating to AE.

### Sampling procedure

A total of 387 questionnaires were filled by the participants. We carried out face to face interviews using structured and pretested questionnaires. Data were collected in randomly selected areas either with or without expected exposure factors such as proximity of people to dogs and other animals.

### Inclusion and exclusion criteria

Individuals with or without any kind of animal association and from any occupational and educational background participated in our study. Children below the age of 15 years were excluded.

### Data collection methods

A questionnaire was created to collect information on sociodemographic variables, and on knowledge, awareness, behaviors, attitudes, landscape epidemiology and disease management concerning AE. Data were collected to establish the components linked with knowledge, attitude, and awareness of AE. Because the condition does not have a distinct local label, the local language was used to describe it to participants, who were asked about their awareness of the disease. These were binary questions with “Yes” or “No” answers. The information was given in narrative text and is shown in Tables 1–6. The questionnaire included KAP items on AE management and control, household information, and dog management and care. Other questions related to risk factors, landscape epidemiology and AE disease management.

**Table 1:** Sociodemographic characteristics of participants.

**Sociodemo-** **graphic factor**	**Characteristic**	**Frequency (*n*)**	**Percentage (%)**
Gender	Male	193	49.9
	Female	194	50.1
Age	+80	2	0.5
	15–20	45	11.6
	21–25	94	24.3
	26–30	40	10.3
	31–35	43	11.1
	36–40	53	13.7
	41–45	52	13.4
	46–50	14	3.6
	51–55	21	5.4
	56–60	5	1.3
	61–65	9	2.3
	71–75	5	1.3
	76–80	4	1.0
Marital status	Married	219	56.6
	Unmarried	168	43.4
Status	Employee	142	36.7
	Other	149	38.5
	Student	96	24.8
Occupation	Animal keeper	7	1.8
	Business	42	10.9
	Butcher	11	2.8
	Farmer	60	15.5
	Government service	60	15.5
	Other	92	23.8
	Professional job	72	18.6
	Shepherd	18	4.7
	Student	24	6.2
Education	Below elementary	11	2.8
	Elementary	15	3.9
	Graduate	15	3.9
	Higher secondary	61	15.8
	No formal education	93	24.0
	Post-graduate	43	11.1
	Secondary	32	8.3
	Undergraduate	117	30.2
Residency	Rural	344	88.8
	Urban	43	11.1
Ethnicity	Brusho	294	76.0
	Others	3	0.8
	Wakhi	90	23.3
Monthly income	200–500 USD	162	41.9
	Above 500 USD	25	6.5
	<200 USD	151	39.0
	No response	49	12.7

**Table 2:** Frequency of knowledge about alveolar echinococcosis.

**Knowledge**	**Characteristic**	**Frequency (*n*)**	**Percentage (%)**
Do you know about parasitic diseases?	Yes	118	30.5
	No	213	55.0
	Maybe	56	14.5
Do you know about zoonosis?	Yes	65	16.8
	No	274	70.8
	Maybe	48	12.4
Do you know about alveolar echinococcosis in human liver and lungs?	Yes	56	14.5
	No	266	68.7
	Maybe	65	16.8
If answer to previous question is yes, symptoms of this disease are what?	Asymptomatic abdominal pain	30	7.8
	Chest pain and cough	35	9.0
	Nausea, chest pain and cough	18	4.7
	Don't know	304	78.6
Ever been infected with this disease?	Yes	10	2.6
	No	377	97.4
Any relative/ family member diagnosed with this particular disease?	Yes	10	2.6
	No	275	71.1
	Maybe	102	26.4
Ever seen this disease in man?	Yes	27	7.0
	No	360	93.0
Which animal may suffer from this disease?	Dog	40	10.3
	Sheep	41	10.6
	Horse	5	1.7
	Other	22	5.7
	Don't know	279	72.1
Ever seen this disease/cyst in animal organs?	Yes	147	38.0
	No	240	62.0
Do you know this disease is transmitted via dog's feces?	Yes	79	20.4
	No	308	79.6
Aware of danger of eating food contaminated with dogs, foxes and jackal feces?	Yes	156	40.3
	No	231	59.7
Do you know playing with dogs may cause infection with disease?	Yes	179	46.3
	No	208	53.7
Do you know rodents (mice, rodents and voles) are intermediate host for the organism causing	Yes	77	19.9
alveolar echinococcosis?	No	310	80.1
Do you know that foxes and jackals are also hosts of the organism causing alveolar echinococcosis?	Yes	58	15.0
	No	329	85.0
Do you know that interaction with foxes and yaks can increase prevalence of disease?	Yes	67	17.3
	No	320	82.7
Do you know you may become infected with disease via contaminated soil?	Yes	134	34.6
	No	253	65.4
Do you know rodents are intermediate hosts of the disease?	Yes	71	18.8
	No	316	81.7
Which of the following is effective treatment/medication for this disease?	Depends on severity of disease	105	27.1
	Surgery	66	17.1
	Drugs	45	11.6
	Don't know	171	44.2

**Table 3:** Attitudes toward alveolar echinococcosis.

**Attitude**	**Scale**	**Frequency (*n*)**	**Percentage (%)**
Do you think you are at risk to get infected with alveolar echinococcosis?	Yes	66	17.1
	No	87	22.5
	Maybe	234	60.5
Do you think that you might get infected with disease by association with	Yes	125	32.3
animals/ infected person?	No	49	12.7
	Maybe	213	55.0
Do you think you might get infected by alveolar echinococcosis by eating Yes	140	36.2	
contaminated food?	No	55	14.2
	Maybe	192	49.6

**Table 4:** Frequency of practices related to alveolar echinococcosis.

**Practices**	**Characteristics**	**Frequency (*n*)**	**Percentage (%)**
Do you have own dogs?	No	345	89.1
	Yes	42	10.8
If yes, then is it tied up?	No	61	15.8
	Maybe	286	73.9
	Yes	40	10.3
How far it is tied up?	<50 m	38	9.8
	Not available	307	79.3
	>50 m	42	10.8
Does your dog have access to kitchen garden or food	No	84	21.7
preparing area?	Not available	285	73.6
	Yes	18	4.7
Does your dog have access to human drinking water?	No	75	19.4
	Not available	285	73.6
	Yes	27	7.0
Do you feed your dog raw offal?	Always	5	1.3
	Often	8	2.1
	Sometimes	22	5.7
	Rarely	14	3.6
	Never	240	62.0
Have you ever noticed rodents on grazing land?	No	157	40.6
	Yes	230	59.4
Do wild animals prey on rodents?	No	154	39.8
	Yes	233	60.2
Do you have wild animals such as dogs, foxes and jackals	No	82	21.2
	Maybe	73	18.9
in your living vicinity?	Yes	232	59.9
If yes, do these wild animals have access to raw offal?	No	90	23.3
	Maybe	150	38.8
	Yes	147	38.0
Do these wild animals have easy access to prey livestock?	No	199	51.4
	Yes	188	48.6
Do you own livestock?	No	86	22.2
	Yes	301	77.8
Do you slaughter livestock at your home?	No	125	32.3
	Yes	262	67.7
Main source of drinking water?	Tap water	331	85.5
	Spring	33	8.5
	Well	23	5.9
Do you boil water before drinking?	Always	65	16.8
	Often	17	4.4
	Sometimes	147	38.0
	Rarely	88	22.7
	Never	70	18.1
Do you wash your hands before eating?	Always	325	84.0
	Often	24	6.2
	Sometimes	21	5.4
	Rarely	6	1.6
	Never	11	2.8
Do you use gloves when handling soil on farmland?	Always	93	24.0
	Often	58	15.0
	Sometimes	89	23.0
	Rarely	89	23.0
	Never	58	15.0
Do you eat leafy vegetables without first washing them?	Always	8	2.1
	Often	26	6.7
	Sometimes	44	11.4
	Rarely	46	11.9
	Never	263	68.0
How do you prepare lettuce leaves as part of salad?	Wash it under running water	283	73.1
	Soak in water	68	17.6
	Wash them with detergent	7	1.8
	Remove the outer leaves and eat the rest	29	7.5

**Table 5:** Landscape epidemiology of alveolar echinococcosis.

**Landscape epidemiology item**	**Characteristic**	**Frequency (*n*)**	**Percentage (%)**
Do environmental factors affect the prevalence of disease?	Yes	108	27.9
	Maybe	245	63.3
	No	34	8.8
Can the landscape influence the level of transmission of an	Yes	161	41.6
infection?	No	226	58.4
Do you know spatial variation of disease risk depends on the area	Yes	138	35.7
of critical habitat and spatial contribution?	No	249	64.3
Does risk of disease depend on connectivity of habitats for vector	Yes	94	24.3
and host?	Maybe	247	63.8
	No	46	11.9
Does landscape control the spatial concentration and diffusion	Yes	72	18.6
of infection risk?	Maybe	254	65.6
	No	61	15.8
Human behavior is an important controlling factor	Strongly disagree	9	2.3
in vector-human contact and in infection.	Disagree	7	1.8
	Neutral	144	37.2
	Agree	131	33.9
	Strongly agree	96	24.8

**Table 6:** Disease management of alveolar echinococcosis.

**Disease management item**	**Characteristic**	**Frequency (*n*)**	**Percentage (%)**
Do hospitals in your area have surgery facilities for AE disease?	Maybe	125	32.3
	No	227	58.7
	Yes	35	9.0
Would you like to receive a disease inspection?	Maybe	62	16.0
	No	68	17.6
	Yes	257	66.4
If you are suffering from the disease, would you take free treatment?	Maybe	66	17.1
	No	52	13.4
	Yes	269	69.5
Would you undergo surgery for this disease if you needed it?	No	79	20.4
	Yes	308	79.6
Do you think vaccination campaigns for animals and humans are required?	Maybe	37	9.6
	No	11	2.8
	Yes	339	87.6
Would like the dogs in your vicinity to be dewormed?	Maybe	69	17.8
	No	38	9.8
	Yes	280	72.4
Do you think that the diet of people as well as animals should be inspected properly (whether	Maybe	42	10.9
infected with *Echinococcosis* eggs or not)?	No	18	4.7
	Yes	327	84.5
Is greater awareness about the impact of the environment on humans and animals needed?	No	29	7.5
	Yes	358	92.5
Proper treatment facilities needed?	No	26	6.7
	Yes	361	93.3
Would you like campaigns on disease counseling and control?	No	38	9.8
	Yes	349	90.2
Proper slaughter houses are needed?	No	28	7.2
	Yes	359	92.8

### Questionnaire design

The questionnaire was designed to gather information on socio-demographics, KAPs, landscape epidemiology and AE disease management. A total of 73 questions were designed, of which 11 were on sociodemographic, 20 on knowledge, 6 on attitudes, 20 on practices, 6 on landscape epidemiology and 11 on disease management.

### Statistical analysis

Data were entered in an MS Excel sheet and a database was established. Statistical analysis was performed using Jamovi 2.2.8 and 95% confidence interval was used to examine the factors involved in the prevalence of AE. The relationships between factors including KAPs, landscape epidemiology and disease management were analyzed by using logistic regression. A statistically significant difference was considered if *P* < 0.05.

### Study variables

Both independent and dependent variables were included in this study. Dependent variables were knowledge about AE; practices that are associated with disease; attitude toward infection and exposure to dogs; landscape epidemiology; and management of AE. Independent variables included were gender, age, marital status, birthplace, number of family members, occupation, education, residency, religion, ethnicity, village and monthly income.

## Results

### Sociodemographic characteristics

A total of 387 questionnaires were filled by participants targeted from the general population of villages in Hunza, Pakistan. The population sample, who were 15 to 80 years, comprised 49.9% (*n* = 193) males and 50.1% (*n* = 194) females. The major ethnic group was Brusho (76.0%) followed by Wakhi (23.3%). About 24.8% of the sample were in direct or indirect contact with livestock, including animal keepers (1.8%), butchers (2.8%), farmers (15.5%) and shepherds (4.7%). Regarding level of education, 45.2% were highly educated (undergraduate, graduate or post-graduate) whereas 30.8% were educated to secondary school level and 24.0% had no formal education. More details are shown in Table 1.

### Knowledge about AE

Nineteen questionnaire items of knowledge were surveyed in KAPs. Among the participants, 55.0% had never heard of any parasitic diseases and 70.8% had never heard of zoonosis. AE was not known by 68.7%, and the majority of participants had never seen the disease either in man or any animal. Of the participants, 79.6% did not know that AE could be transmitted *via* dog feces, and 59.7% were unaware of the danger of eating food contaminated with dog, fox or jackal feces. The majority of participants (80.1%) did not know about the host of *E. multilocularis*. More details are shown in Table 2.

### Attitudes toward AE

Six KAP questions concerned the attitude of the participants toward AE. Of the participants, 60.5% of the participants thought that they might be at risk of infection with AE, and 32.3% believed that they could become infected with the disease by association with animals or an infected person. Being infected with the disease by eating contaminated food was a belief expressed by 36.2%. More details are given in Table 3.

### Practices related to AE

Among the participants, 10.8% owned a dog and 15.8% did not tie up their dogs; 21.7% of individuals who owned dogs did not give them access to the kitchen garden and 19.4% did not give excess to human drinking water, respectively. Interestingly, 59.4% of the participants noticed rodents on grazing land and 60.2% of the participants thought that wild animals prey on rodents. In addition, 59.9% of the participants reported seeing wild animals (foxes) in their locality. Participants believed that these wild animals had easy access to raw offal (38.0%) and that they preyed on livestock (48.6%). The majority of the participants (77.8%) kept livestock. Glaciers were the main source of drinking water for the participants (85.5%) and 16.8% always boiled water before drinking. Use of gloves when handling soil was reported by 24.0% of the participants and the majority (68.0%) reported never eating leafy vegetables without washing them. More details are given in Table 4.

### Landscape epidemiology of AE

Six KAP questions were about landscape epidemiology of AE. Of the participants, 27.9% knew about the effect of environmental factors on the prevalence of AE. Only 41.6% of the participants knew that landscape influenced the level of transmission of infection. Another 35.7 and 24.3% knew about the spatial variation of the disease and the connectivity of habitat with intermediate and final hosts, respectively. Only 18.6% knew about landscape management, spatial concentration and infection diffusion risk. The details are shown in Table 5.

### Disease management of AE

Eleven questions in the KAP study were about management of AE. Only 9.0% of the participants reported that hospitals in their area had surgical facilities to treat the disease. Most of the participants indicated they would like to be screened for disease (66.4%) and receive free treatment (69.5%). The majority (87.6%) thought that vaccination campaigns for animals and humans are required and (72.4%) of participants indicated they would like all dogs in the community to be dewormed. A large number of the participants (84.5%) indicated that they would like a proper diet inspection (whether food they are eating is at risk of contamination with *E. multilocularis* eggs) of both humans and animals. According to 92.5% of the participants, awareness about the disease is needed and the majority (93.3%) also thought that proper veterinary and sanitation facilities were required in their areas. Details are shown in Table 6.

### Statistical analysis of the relationship between KAPs, landscape epidemiology and disease management and the population sociodemographic characteristics

Using logistic regression, we assessed the association between the independent sociodemographic factors of age, gender, religion, ethnicity, education, marital status, occupation and income and the dependent factors of KAPs, landscape epidemiology and AE disease management, to determine the factors most associated with this disease.

The analysis results indicated that the study variables (KAPs, landscape epidemiology and AE disease management) had a moderate dependency on the defined sociodemographic aspects (age, gender, religion, ethnicity, education, marital status, occupation and income) of the study (Tables 7–11).

**Table 7:** Association between sociodemographic factors and knowledge.

**Variable**	**Characteristics**	**Knowledge status**		**Estimate**	**Standard error**	***Z*-value**	***P*-value**	**Odds ratio**	***R* ^2^_McF_**
		**Good**	**Poor**						
Gender	Male	85	109	−0.140	0.206	−0.677	0.498	0.870 (−0.543–0.264)	8.71e-4
	Female (Base)	78	115	NA	NA	NA	NA	NA	
Age	15–20 (Base)	20	25	NA	NA	NA	NA	NA	0.0134
	21–25	40	54	0.0770	0.365	0.211	0.833	1.080 (−0.639–0.793)	
	26–30	17	23	0.0791	0.439	0.180	0.857	1.082 (−0.780–0.939)	
	31–35	15	28	0.4010	0.439	0.914	0.361	1.493 (−0.459–1.261)	
	36–40	23	30	0.0426	0.408	0.104	0.917	1.043 (−0.758–0.843)	
	41–45	25	27	−0.1462	0.409	−0.358	0.721	0.864 (−0.947–0.655)	
	46–50	5	9	0.3646	0.633	0.576	0.565	1.440 (−0.877–1.606)	
	51–55	9	12	0.0645	0.533	0.121	0.904	1.067 (−0.981–1.110)	
	56–60	2	3	0.1823	0.961	0.190	0.850	1.200 (−1.701–2.066)	
	61–65	1	8	1.8563	1.102	1.684	0.092	6.400 (−0304–4.017)	
	71–75	3	2	−0.6286	0.961	−0.654	0.513	0.533 (−2.512–1.255)	
	76–80	2	2	−0.2231	1.044	−0.214	0.831	0.800 (−2.269–1.823)	
	80+	1	1	−0.2231	1.446	−0.154	0.877	0.800 (−3.057–2.610)	
Education	No formal education	35	58	0.3228	0.642	0.5026	0.615	1.381	0.0146
	Below elementary (base)	5	6	NA	NA	NA	NA	NA	
	Elementary	8	7	−0.3159	0.797	−0.3965	0.692	0.729 (−1.877–1.245)	
	Secondary	18	14	−0.4336	0.703	−0.6172	0.537	0.648 (−1.811–0.943)	
	Higher secondary	27	34	0.0482	0.658	0.0732	0.942	1.049 (−1.242–1.338)	
	Undergraduate	50	67	0.1103	0.634	0.1741	0.862	1.117 (−1.132–1.352)	
	Graduate	3	12	1.2040	0.885	1.3603	0.174	3.333 (−0.531–2.939)	
	Post-graduate	17	26	0.2426	0.681	0.3561	0.722	1.275 (−1.09–1.578)	
Residency	Rural (base)	143	201	NA	NA	NA	NA	NA	7.22e-4
	Urban	20	23	−0.201	0.325	−0.618	0.537	0.818 (−0.837–0.436)	
Marital status	Married (base)	92	127	NA	NA	NA	NA	NA	4.73e-6
	Unmarried	71	97	−0.0104	0.208	−0.0499	0.960	0.990 (−0.4174–0.397)	
Status	Employee	58	84	NA	NA	NA	NA	NA	0.00160
	Student	38	38	0.0525	0.270	0.195	0.846	1.054 (−0.4760–0.581)	
	Other	67	82	−0.1683	0.237	−0.710	0.478	0.845 (−0.6333–0.297)	
Monthly income	<40 k	59	92	0.271	0.230	1.180	0.238	1.31 (−0.179–0.721)	0.00284
	40–100 k (Base)	74	8	NA	NA	NA	NA	NA	
	Above 100 k	10	15	0.232	0.438	0.531	0.596	1.26 (−0.626–1.090)	
	No response	20	29	0.198	0.331	0.600	0.549	1.22 (−0.450–0.846)	
Birthplace	Aliabad	17	16	NA	NA	NA	NA	NA	0.0686
	Altit	3	12	1.4469	0.733	1.97268	0.049^*^	4.250 (0.00933–2.885)	
	Attabad	0	4	16.6267	1199.772	0.01386	0.989	1.66e+7 (−2334.88402–2368.137)	
	Chipurson	3	3	0.0606	0.888	0.06829	0.946	1.063 (−1.67921–1.800)	
	Dorkhun	5	2	−0.8557	0.906	−0.94416	0.345	0.425 (−2.63192–0.92)	
	Ganish	9	11	0.2613	0.569	0.45951	0.646	1.299 (−0.85321–1.376)	
	Garelth	4	9	0.8716	0.695	1.25480	0.210	2.391 (−0.48979–2.233)	
	Ghulkin	2	5	0.9769	0.906	1.07795	0.281	2.656 (−0.79934–2.753)	
	Gilgit	10	11	0.1559	0.559	0.27906	0.780	1.169 (−0.93925–1.251)	
	Gojal	1	0	−16.5054	2399.545	−0.00688	0.995	6.79e-8 (−4719.52672–4686.512)	
	Gulmit	5	10	0.7538	0.649	1.16127	0.246	2.125 (−0.51843–2.026)	
	Hassanabad	5	9	0.6484	0.658	0.98603	0.324	1.913 (−0.64046–1.937)	
	Hunza	31	51	0.5585	0.416	1.34194	0.180	1.748 (−0.25720–1.374)	
	Hussaini	5	5	0.0606	0.722	0.08396	0.933	1.063 (−1.35452–1.476)	
	Hyderabad	5	11	0.8491	0.642	1.32245	0.186	2.338 (−0.40932–2.107)	
	Islamabad	0	1	16.6267	2,399.545	0.00693	0.994	1.66e+7 (−4686.39459–4719.648)	
	Karachi	0	3	16.6267	1,385.378	0.01200	0.990	1.66e+7 (−2698.66397–2731.917)	
	Karimabad	10	10	0.0606	0.567	0.10695	0.915	1.063 (−1.05039–1.172)	
	Khudabad	1	5	1.6701	1.149	1.45287	0.146	5.313 (−0.58289–3.923)	
	Khyber	6	5	−0.1217	0.699	−0.17421	0.862	0.885 (−1.49086–1.247)	
	Misgar	2	2	0.0606	1.059	0.05725	0.954	1.063 (−2.0148–2.136)	
	Morkhun	2	0	−16.5054	1696.734	−0.00973	0.992	6.79-8 (−3342.04372–3309.033)	
	Murtazabad	11	7	−0.3914	0.596	−0.65676	0.511	0.676 (−1.55929–0.777)	
	Nasirabad	1	1	0.0606	1.456	0.04162	0.967	1.063 (−2.79402–2.915)	
	Nazimabad	0	1	16.6267	2,399.545	0.00693	0.994	1.66e+7 (−4686.39459–4719.648)	
	Passu	3	2	−0.3448	0.977	−0.35293	0.724	0.708 (−2.25985–1.570)	
	Shimshal	7	9	0.3119	0.613	0.50920	0.611	1.366 (−0.88876–1.513)	
	Shishkat	6	12	0.7538	0.609	1.23698	0.216	2.125 (−0.44056–1.948)	
	Sost	9	6	−0.3448	0.632	−0.54585	0.585	0.708 (−1.58304–0.893)	
	Sultanabad	0	1	16.6267	2,399.545	0.00693	0.994	1.66e+7 (−4686.39459–4719.648)	
Occupation	Farmer	23	37	0.188	0.809	0.2322	0.816	1.207 (−1.40–1.773)	
	Animal keeper (base)	3	4	NA	NA	NA	NA	NA	
	Butcher	8	3	−1.269	1.021	−1.2429	0.214	0.281 (−3.27–0.732)	
	Shepherd	7	11	0.164	0.904	0.1818	0.856	1.179 (−1.61–1.936)	
	Business	18	24	1.68e−15	0.825	2.04e-15	1.000	1.000 (−1.62–1.617)	
	Professional job	33	39	−0.121		−0.1509	0.880	0.886 (−1.69–1.446)	
	Government service	24	36	0.118	0.808	0.1458	0.884	1.125 (−1.47–1.701)	
	Other	37	55	0.109	0.793	0.1371	0.891	1.115 (−1.45–1.663)	
	Housewife	1	0	−13.854	535.412	−0.0259	0.979	9.62e−7 (−1063.24–1035.534)	
	Student	9	15	0.223	0.872	0.2558	0.798	1.250 (−1.49–1.933)	
Religion	Muslim (base)	163	223	NA	NA	NA	NA	NA	0.00208
	Other	0	1	13.253	535.411	0.0248	0.980	569,574.53 (−1036.134–1062.639)	
Ethnicity	Brusho (base)	121	173	NA	NA	NA	NA	NA	0.0102
	Wakhi	39	51	−0.0892	0.244	−0.3665	0.714	0.915 (−0.566–0.388)	
	Not available	3	0	−14.9236	509.652	−0.0293	0.977	3.30e-7 (−1013.823–983.976)	
Village	Aliabad (base)	14	21	NA	NA	NA	NA	NA	0.0784
	Altit	8	12	−2.24e-15	0.572	3.92e-15	1.000	1.000 (−1.121–1.1214)	
	Attabad	0	2	16.1606	1,696.734	0.00952	0.992	1.04e+7 (−3309.378–3341.6989)	
	Chipurson	3	3	−0.4055	0.886	−0.45743	0.647	0.667 (−2.143–1.3319)	
	Dorkhun	11	5	−1.1939	0.640	−1.86469	0.062	0.303 (−2.449–0.0610)	
	Ganish	9	14	0.0364	0.549	0.06622	0.947	1.037 (−1.040–1.1127)	
	Garelth	4	9	0.4055	0.693	0.58514	0.558	1.500 (−0.953–1.7636)	
	Ghulkin	3	5	0.1054	0.808	0.13044	0.896	1.111 (−1.478–16884)	
	Gilgit	2	3	−2.59e-15	0.976	−2.65e-15	1.000	1.000 (−1.913–1.9127)	
	Gulmit	4	10	0.5108	0.685	0.74587	0.456	1.667 (−0.831–1.8531)	
	Hassanabad	4	10	0.5108	0.685	0.74587	0.456	1.667 (−0.831–1.8531)	
	Hunza	10	13	−0.1431	0.544	−0.26304	0.793	0.867 (−1.209–0.9232)	
	Hussaini	6	5	−0.5878	0.697	−0.84339	0.399	0.556 (−1.954–0.7782)	
	Hyderabad	6	20	0.7985	0.579	1.37814	0.168	2.222 (−0.337–1.9341)	
	Jutial	1	0	−16.9715	2399.545	−0.00707	0.994	4.26e-8 (−4719–993–4686.0497)	
	Karimabad	13	12	−0.4855	0.528	−0.91867	0.358	0.615 (−1.521–0.5503)	
	Khanabad	1	3	0.6931	1.205	0.57516	0.565	2.000 (−1.669–3.0552)	
	Khudabad	3	5	0.1054	0.808	0.13044	0.896	1.111 (−1478–1.6884)	
	Khyber	7	4	−0.9651	0.715	−1.34887	0.177	0.381 (−2.367–0.4372)	
	Mayoon	0	1	16.1606	2,399.545	0.00673	0.995	1.04e+7 (−4686.861–4718.1819)	
	Misgar	2	5	0.5108	0.905	0.56444	0.572	1.667 (−1.263–2.2846)	
	Morkhun	2	0	−16.9715	1,696.734	−0.01000	0.992	4.26e-8 (−3342.510–3308.5667)	
	Murtazabad	18	13	−0.7309	0.502	−1.45733	0.145	0.481 (−1.714–0.2521)	
	Nasirabad	3	1	−1.5041	1.205	−1.24804	0.212	0.222 (−3.866–0.8580)	
	Nazimabad	0	2	16.1606	1,696.734	0.00952	0.992	1.04e+7 (−3309.378–3341.6989)	
	Nomal	1	1	−0.4055	1.456	−0.27854	0.781	0.667 (−3.259–2.4476)	
	Oshikhandass	1	0	−16.9715	2,399.545	−0.00707	0.994	4.26e-8 (−4719.993–4686.0497)	
	Passu	3	2	−0.8109	0.976	−0.83096	0.406	0.444 (−2.724–1.1018)	
	Rahimabad	0	2	16.1606	1,696.734	0.00952	0.992	1.04e+7 (−3309.378–3341.6989)	
	Shimshal	7	15	0.3567	0.573	0.62224	0.534	1.429 (−0.767–1.4801)	
	Shishkat	8	19	0.4595	0.545	0.84367	0.399	1.583 (−0.608–1.5271)	
	Sost	8	6	−0.6931	0.641	−1.08157	0.279	0.500 (−1.949–0.5629)	
	Sultanabad	1	1	−0.4055	1.456	−0.27854	0.781	0.667 (−3.259–2.4476)	
Number of family members	2	0	1	NA	NA	NA	NA	NA	0.0254
	3	4	4	−14.6	883	−0.0165	0.987	4.72e-7 (−1745–1716)	
	4	16	21	−14.3	883	−0.0162	0.987	6.20e-7 (−1744–1716)	
	5	35	47	−14.3	883	−0.0162	0.987	6.34e-7 (−1744–1716)	
	6	41	46	−14.5	883	−0.0164	0.987	5.30e-7 (−1745–1716)	
	7	26	39	−14.2	883	−0.0160	0.987	7.08e-7 (−1744–1716)	
	8	12	27	−13.8	883	−0.0156	0.988	1.06e-6 (−1744–1716)	
	9	12	16	−14.3	883	−0.0162	0.987	6.29e-7 (−1744–1716)	
	10	9	6	−15.0	883	−0.0170	0.986	3.15e-7 (−1745–1715)	
	11	1	3	−13.5	883	−0.0153	0.988	1.42e-6 (−1744–1717)	
	12	1	7	−12.6	883	−0.0143	0.989	3.30e-6 (−1743–1718)	
	13	0	1	−9.69e-9	1,248	−7.76e-12	1.000	1.000 (−2447–2447)	
	14	2	1	−15.3	883	−0.0173	0.986	2.36e-7 (−1745–1715)	
	15	2	2	−14.6	883	−0.0165	0.987	4.72e-7 (−1745–1716)	
	16	1	1	−14.6	883	−0.0165	0.987	4.72e−7 (−1745–1716)	
	17	0	1	−9.75e-9	1,248	−7.81e-12	1.000	1.000 (−2447–2447)	
	18	1	1	−14.6	883	−0.0165	0.987	4.72e-7 (−1745–1716)	

* indicates the values which are significant.

**Table 8:** Association between attitudes and sociodemographic factors.

**Variable**	**Characteristic**	**Attitude status**		**Estimate**	**Standard error**	***Z*-value**	***P*-value**	**Odds ratio**	***R* ^2^_McF_**
		**Good**	**Poor**						
Gender	Male	**128**	**66**	−0.0536	0.214	−0.251	0.802	0.948 (−0.472–0.365)	1.26e-4
	Female	125	68	NA	NA	NA	NA	NA	
	15–20	33	12	NA	NA	NA	NA	NA	0.0637
	21–25	52	42	0.798	0.396	2.0161	0.044	2.221 (0.0222–1.574)	
	26–30	34	6	−0.723	0.557	−1.2991	0.194	0.485 (−1.8138–0.368)	
	31–35	21	22	1.058	0.455	2.3273	0.020^*^	2.881 (0.1670–1.949)	
	36–40	36	17	0.261	0.447	0.5839	0.559	1.299 (−0.6157–1.138)	
	41–45	35	17	0.289	0.448	0.6456	0.519	1.336 (−0.5893–1.168)	
	46–50	8	6	0.724	0.637	1.1371	0.255	2.063 (−0.5239–1.972)	
	51–55	18	3	−0.780	0.709	−1.1005	0.271	0.458 (−2.1696–0.609)	
	56–60	2	3	1.417	0.973	1.4562	0.145	4.125 (−0.4902–3.324)	
	61–65	4	5	1.235	0.751	1.6447	0.100	3.437 (−0.2367–2.709)	
	71–75	4	1	−0.375	1.168	−0.3209	0.748	0.687 (−2.6634–1.914)	
	76–80	4	0	−14.554	727.699	−0.0200	0.984	4.78e-7 (−1440.8180–1411.709)	
	80+	2	0	−14.554	1,029.122	−0.0141	0.989	4.78e-7 (−2031.5956–2002.487)	
Education	No formal education	67	26	0.557	0.815	0.684	0.494	1.746 (−1.040–2.1552)	0.0165
	Below elementary	9	2	NA	NA	NA	NA	NA	
	Elementary	8	7	1.371	0.938	1.462	0.144	3.938 (−0.467–3.2081)	
	Secondary	18	14	1.253	0.859	1.458	0.145	3.500 (−0.431–2.9366)	
	Higher secondary	40	21	0.860	0.827	1.040	0.298	2.363 (−0.761–2.4804)	
	Undergraduate	79	38	0.772	0.806	0.958	0.338	2.165 (−0.808–2.3525)	
	Graduate	8	7	1.371	0.938	1.462	0.144	3.938 (−0.467–3.2081)	
	Post–graduate	24	19	1.270	0.840	1.513	0.130	3.562 (−0.376–2.9166)	
Residency	Rural (base)	224	120	NA	NA	NA	NA	NA	1.84e-4
	Urban	29	14	−0.104	0.345	−0.302	0.765	0.901 (−0.779–0.571)	
Marital status	Married (base)	144	75	NA	NA	NA	NA	NA	6.40e-5
	Unmarried	109	59	0.0385	0.215	0.179	0.858	1.039 (−0.384–0.461)	
Status	Employee (base)	87	55	NA	NA	NA	NA	NA	0.00729
	Student	60	36	−0.0523	0.272	−0.192	0.848	0.949 (−0.586–0.4814)	
	Other	106	43	−0.4437	0.250	−1.777	0.076	0.642 (−0.933–0.0458)	
Monthly income	<40	103	48	−0.153	0.240	−0.636	0.525	0.858 (−0.623–0.318)	0.00249
	40–100 k (base)	105	57	NA	NA	NA	NA	NA	
	Above 100 k	15	10	0.205	0.440	0.467	0.641	1.228 (−0.657–1.068)	
	No response	30	19	0.154	0.336	0.459	0.647	1.167 (−0.505–0.813)	
Birthplace	Aliabad (base)	20	13	NA	NA	NA	NA	NA	0.0721
	Altit	9	6	0.0253	0.636	0.0398	0.968	1.0256 (−1.222–1.272)	
	Attabad	2	2	0.4308	1.062	0.4058	0.685	1.5385 (−1.650–2.511)	
	Chipurson	5	1	−1.1787	1.152	−1.0232	0.306	0.3077 (−3.436–1.079)	
	Dorkhun	5	2	−0.4855	0.909	−0.5339	0.593	0.6154 (−2.268–1.297)	
	Ganish	9	11	0.6315	0.574	1.1010	0.271	1.8803 (−0.493–1.756)	
	Garelth	12	1	−2.0541	1.100	−1.8672	0.062	0.1282 (−4.210–0.102)	
	Ghulkin	4	3	0.1431	0.843	0.1698	0.865	1.1538 (−1.509–1.795)	
	Gilgit	15	6	−0.4855	0.600	−0.8089	0.419	0.6154 (−1.662–0.691)	
	Gojal	0	1	15.9969	1,455.398	0.0110	0.991	8.86e+6 (−2836.530–2868.524)	
	Gulmit	9	6	0.0253	0.636	0.0398	0.968	1.0256 (−1.222–1.272)	
	Hassanabad	9	5	−0.1570	0.662	−0.2372	0.812	0.8547 (−1.454–1.140)	
	Hunza	50	32	−0.0155	0.422	−0.0367	0.971	0.9846 (−0.843–0.812)	
	Hussaini	6	4	0.0253	0.737	0.0343	0.973	1.0256 (−1.420–1.470)	
	Hyderabad	12	4	−0.6678	0.678	−0.9844	0.325	0.5128 (−1.998–0.662)	
	Islamabad	1	0	−15.1353	1,455.398	−0.0104	0.992	2.67e-7 (−2867.662–2837.392)	
	Karachi	1	2	1.1239	1.276	0.8812	0.378	3.0769 (−1.376–3.624)	
	Karimabad	16	4	−0.9555	0.663	−1.4414	0.149	0.3846 (−2.255–0.344)	
	Khudabad	3	3	0.4308	0.891	0.4836	0.629	1.5385 (−1.315–2.177)	
	Khyber	6	5	0.2485	0.703	0.3537	0.724	1.2821 (−1.129–1.625)	
	Misgar	3	1	−0.6678	1.208	−0.5527	0.581	0.5128 (−3.036–1.701)	
	Morkhun	2	0	−15.1353	1,029.122	−0.0147	0.988	2.67e-7 (−2032.176–2001.906)	
	Murtazabad	12	6	−0.2624	0.614	−0.4273	0.669	0.7692 (−1.466–0.941)	
	Nasirabad	1	1	0.4308	1.458	0.2954	0.768	1.5385 (−2.428–3.289)	
	Nazimabad	1	0	−15.1353	1,455.398	−0.0104	0.992	2.67e-7 (−2867.662–2837.392)	
	Passu	3	2	0.0253	0.980	0.0258	0.979	1.0256 (−1.895–1.946)	
	Shimshal	12	4	−0.6678	0.678	−0.9844	0.325	0.5128 (−1.998–0.662)	
	Shishkat	17	1	−2.4024	1.089	−2.2063	0.027^*^	0.0905 [−4.537– (−0.268)]	
	Sost	8	7	0.2973	0.628	0.4731	0.636	1.3462 (−0.934–1.529)	
	Sultanabad	0	1	15.9969	1,455.398	0.0110	0.991	8.86e+6 (−2836.530)	
Occupation	Farmer	45	15	−1.386	0.820	−1.6908	0.091	0.250 (−2.99–0.221)	0.0202
	Animal keeper (base)	3	4	NA	NA	NA	NA	NA	
	Butcher	5	6	−0.105	0.975	−0.1081	0.914	0.900 (−2.02–1.805)	
	Shepherd	12	6	−0.981	0.913	−1.0744	0.283	0.375 (−2.77–0.808)	
	Business	24	18	−0.575	0.825	−0.6974	0.486	0.562 (−2.19–1.042)	
	Professional job	48	24	−0.981	0.804	−1.2205	0.222	0.375 (−2.56–0.594)	
	Government service	41	19	−1.057	0.813	−1.3005	0.193	0.348 (−2.65–0.536)	
	Other	56	36	−0.730	0.793	−0.9199	0.358	0.482 (−2.28–0.825)	
	Student	18	6	−1.386	0.898	−1.5446	0.122	0.250 (−3.15–0.373)	
	Housewife	1	0	−13.854	535.412	−0.0259	0.979	9.62e-7 (−1063.24–1035.534)	
Religion	Muslim (base)	252	134	NA	NA	NA	NA	NA	0.00171
	Other	1	0	−12.934	535.411	−0.0242	0.981	2.41e-6 (−1062.321)	
Ethnicity	Brusho	196	98	NA	NA	NA	NA	NA	0.00780
	Wakhi	54	36	0.288	0.248	1.1591	0.246	1.333 (−0.199–0.774)	
	Not available	3	0	−13.873	509.652	−0.0272	0.978	9.44e-7 (−1012.773–985.027)	
Village	Aliabad	18	17	NA	NA	NA	NA	NA	0.090
	Altit	12	8	−0.3483	0.568	−0.61313	0.540	0.7059 (−1.462–0.7651)	
	Attabad	2	0	−17.5089	2,797.442	−0.00626	0.995	2.49e-8 (−5500.394–5465.3766)	
	Chipurson	5	1	−1.5523	1.146	−1.35397	0.176	0.2118 (−3.799–0.6947)	
	Dorkhun	10	6	−0.4537	0.617	−0.73493	0.462	0.6353 (−1.664–0.7562)	
	Ganish	12	11	−0.0299	0.537	−0.05557	0.956	0.9706 (−1.083–1.0231)	
	Garelth	12	1	−2.4277	1.094	−2.21834	0.027^*^	0.0882 [−4.573– (−0.2828)]	
	Ghulkin	4	4	0.0572	0.784	0.07292	0.942	1.0588 (−1.479–1.5934)	
	Gilgit	5	0	−17.5089	1,769.258	−0.00990	0.992	2.49e-8 (−3485.190–3450.1724)	
	Gulmit	8	6	−0.2305	0.637	−0.36177	0.718	0.7941 (−1.479–1.0184)	
	Hassanabad	10	4	−0.8591	0.681	−1.26073	0.207	0.4235 (−2.195–0.4765)	
	Hunza	18	5	−1.2238	0.608	−2.01205	0.044^*^	0.2941 [−2.416– (−0.0317)]	
	Hussaini	6	5	−0.1252	0.694	−0.18046	0.857	0.8824 (−1.485–1.2342)	
	Hyderabad	16	10	−0.4128	0.526	−0.78459	0.433	0.6618 (−1.444–0.6185)	
	Jutial	0	1	17.6232	3,956.180	0.00445	0.996	4.50e+7 (−7736.348–7771.5942)	
	Karimabad	15	10	−0.3483	0.530	−0.65701	0.511	0.7059 (−1.387–0.6907)	
	Khanabad	4	0	−17.5089	1,978.090	−0.00885	0.993	2.49e-8 (−3894494–3859.4766)	
	Khudabad	4	4	0.0572	0.784	0.07292	0.942	1.0588 (−1.479–1.5934)	
	Khyber	6	5	−0.1252	0.694	−0.18046	0.857	0.8824 (−1.485–1.2342)	
	Mayoon	1	0	−17.5089	3,956.180	−0.00443	0.996	2.49e-8 (−771.480–7736.4621)	
	Misgar	4	3	−0.2305	0.835	−0.27598	0.783	0.7941 (−1.868–1.4066)	
	Morkhun	2	0	−17.5089	2,797.442	−0.00626	0.995	2.49e-8 (−5500.394–5465.3766)	
	Murtazabad	23	8	−0.9989	0.532	−1.87817	0.060	0.3683 (−2.041–0.0435)	
	Nasirabad	1	3	1.1558	1.203	0.96057	0.337	3.1765 (−1.202–3.5140)	
	Nazimabad	2	0	−17.5089	2,797.442	−0.00626	0.995	2.49e-8 (−5500.394–5465.3766)	
	Nomal	1	1	0.0572	1.454	0.03931	0.969	1.0588 (−2.793–2.9071)	
	Oshikhandass	1	0	−17.5089	3,956.180	−0.00443	0.996	2.49e-8 (−7771.480–7736.4621)	
	Passu	3	2	−0.3483	0.974	−0.35779	0.721	0.7059 (−2.256–1.5597)	
	Rahimabad	0	2	17.6232	2,797.442	0.00630	0.995	4.50e+7 (−5465.262–5500.5087)	
	Shimshal	16	6	−0.9237	0.586	−1.57589	0.115	0.3971 (−2.072–0.2251)	
	Shishkat	22	5	−1.4244	0.600	−2.37463	0.018	0.2406 (−2.600– (−0.2487)	
	Sost	8	6	−0.2305	0.637	−0.36177	0.718	0.7941 (−1.479–1.0184)	
	Sultanabad	2	0	−17.5089	2,797.442	−0.00626	0.995	2.49e-8 (5500.394–5465.3766)	
Number of family members	2	0	1	NA	NA	NA	NA	NA	0.0190
	3	5	3	−16.1	1,455	−0.0110	0.991	1.04e0–7 (−2869–2836)	
	4	23	14	−16.1	1,455	−0.0110	0.991	1.06e0–7 (−2869–2836)	
	5	52	30	−16.1	1,455	−0.0111	0.991	1.00e0–7 (−2869–2836)	
	6	59	28	−16.3	1,455	−0.0112	0.991	8.24e0–8 (−2869–2836)	
	7	43	22	−16.2	1,455	−0.0112	0.991	8.89e0–8 (−2869–2836)	
	8	27	12	−16.4	1,455	−0.0113	0.991	7.72e0–8 (−2869–2836)	
	9	18	10	−16.2	1,455	−0.0111	0.991	9.65e0–8 (−2869–2836)	
	10	8	7	−15.7	1,455	−0.0108	0.991	1.52e0–7 (−2868–2837)	
	11	3	1	−16.7	1,455	−0.0115	0.991	5.79e0–8 (−2869–2836)	
	12	6	2	−16.7	1,455	−0.0115	0.991	5.79e0–8 (−2869–2836)	
	13	1	0	−31.1	2,058	−0.0151	0.988	3.02e-14 (−4065–4003)	
	14	3	0	−31.1	1,681	−0.0185	0.985	3.02e-14 (−3325–3263)	
	15	2	2	−15.6	1,455	−0.0107	0.991	1.74e0–7 (−2868–2837)	
	16	1	1	−15.6	1,455	−0.0107	0.991	1.74e0–7 (−2868–2837)	
	17	1	0	−31.1	2,058	−0.0151	0.988	3.02e-14 (−4065–4003)	
	18	1	1	−15.6	1,455	−0.0107	0.991	1.74e0–7 (−2868–2837)	

* indicates the values which are significant.

Standard error: Provides the estimated standard deviation of the distribution of coefficients; *P*-value: Indicates the probability of observing the coefficient value, or more extreme, if the null hypothesis is correct; Z-value: Is computed by estimate/std_error. Odd ratio: Compares the odd of two events; R^2^McF: The parameter estimates those values which maximize the likelihood of the data which have been observed; Estimate: Predicts the value of the dependent variable when the values for the independent variables are given.

**Table 9:** Association between practices and sociodemographic factors.

**Variable**	**Characteristic**	**Practices status**		**Estimate**	**Standard error**	***Z*-value**	***P*-value**	**Odds ratio**	**R^2^_McF_**
		**Good**	**Poor**						
Gender	Male	0.770	0.770	0.770	0.770	0.770	0.770	0.770 (−0.601–0.204)	0.770
	Female (Base)	104	89	NA	NA	NA	NA	NA	
Age	15–20 (Base)	21	24	NA	NA	NA	NA	NA	0.0332
	21–25	50	44	−0.261	0.363	−0.7193	0.472	0.770 (−0.973–0.451)	
	26–30	23	17	−0.436	0.438	−0.9957	0.319	0.647 (−1.294–0.422)	
	31–35	28	15	−0.758	0.438	−1.7307	0.084	0.469 (−1.616–0.100)	
	36–40	31	22	−0.476	0.409	−1.1660	0.244	0.621 (−1.277–0.324)	
	41–45	31	21	−0.523	0.411	−1.2716	0.204	0.593 (−1.329–0.283)	
	46–50	6	8	0.154	0.617	0.2498	0.803	1.167 (1.056–1.364)	
	51–55	17	4	−1.580	0.631	−2.5048	0.012	0.206 (−2.817–0.344)	
	56–60	4	1	−1.520	1.157	−1.3133	0.189	0.219 (−3.788–0.748)	
	61–65	3	6	0.560	0.768	0.7290	0.466	1.750 (−0.945–2.064)	
	71–75	2	3	0.272	0.961	0.2831	0.777	1.312 (−6.11–2.155)	
	76–80	2	2	−0.134	1.044	−0.1279	0.898	0.875 (−2.179–1.912)	
	80+	0	2	14.433	624.194	0.0231	0.982	1.85e+6 (−1.208.965–1237.830)	
Education	No formal education	58	35	0.0545	0.662	0.0823	0.934	1.056 (−1.244–1.353)	0.0199
	Below elementary (base)	7	4	NA	NA	NA	NA	NA	
	Elementary	8	7	0.4261	0.813	0.5242	0.600	1.531 (−1.165–2.019)	
	Secondary	12	20	1.0704	0.725	1.4757	0.140	2.917 (0.351–2.492)	
	Higher secondary	35	26	0.2624	0.678	0.3869	0.699	1.300 (−1.067–1.592)	
	Undergraduate	62	55	0.4398	0.654	0.6729	0.501	1.552 (−0.841–1.721)	
	Graduate	12	3	−0.8267	0.900	−0.9188	0.358	0.438 (−2.590–0.937)	
	Post-graduate	24	19	0.3260	0.698	0.4671	0.640	1.385 (1.042–1.694)	
Residency	Rural (base)	199	19	NA	NA	NA	NA	NA	0.00542
	Urban	19	24	0.550	0.326	1.69	0.091	1.734 (−0.0886–1.189)	
Marital status	Married (base)	136	83	NA	NA	NA	NA	NA	0.0129
	Unmarried	82	86	0.541	0.208	2.60	0.009^*^	1.718 (0.134–0.949)	
Status	Employee (base)	80	62	NA	NA	NA	NA	NA	0.00484
	Student	48	48	0.255	0.265	0.961	0.336	1.290 (−0.265–0.7745)	
	Other	90	59	−0.167	0.238	−0.703	0.482	0.846 (0.634–0.2993)	
Monthly income	<40	81	70	0.332	0.230	1.45	0.148	1.394 (−0.118–0.782)	0.00686
	40–100 k (base)	100	62	NA	NA	NA	NA	NA	
	Above 100 k	12	13	0.558	0.432	1.29	0.196	1.747 (−0.288–1.404)	
	No response	25	24	0.437	0.328	1.33	0.183	1.548 (0.206–1.081)	
Birthplace	Aliabad (base)	24	9	NA	NA	NA	NA	NA	0.156
	Altit	4	11	1.992	0.703	2.83568	0.005^*^	7.333 (0.6153–3.370)	
	Attabad	0	4	18.547	1,978.090	0.00938	0.993	1.13e+8 (−3858.43873895.532)	
	Chipurson	0	6	18.547	1,615.104	0.01148	0.991	1.13e+8 (3146.9986–3184.092)	
	Dorkhun	2	5	1.897	0.923	2.05436	0.040^*^	6.667 (0.0872–3.7071)	
	Ganish	13	7	0.362	0.610	0.59273	0.553	1.436 (−0.8345–1.558)	
	Garelth	11	2	−0.724	0.862	−0.83945	0.401	0.485 (−24141–0.966)	
	Ghulkin	7	0	−16.585	1,495.296	−0.01109	0.991	6.27e-8 (−2947.3109–2914.140)	
	Gilgit	14	7	0.288	0.606	0.47483	0.635	1.333 (−0.8998–1.475)	
	Gojal	1	0	−16.585	3,956.180	−0.00419	0.997	6.27e-8 (−7770.5562–7737.386)	
	Gulmit	10	5	0.288	0.673	0.42753	0.669	1.333 (−1.0312–1.607)	
	Hassanabad	12	2	−0.811	0.858	−0.94517	0.345	0.444 (−2.4925–0.871)	
	Hunza	36	46	1.226	0.450	2.72572	0.006 ^*^	3.407 (0.3444–2.107)	
	Hussaini	3	7	1.828	0.793	2.30511	0.021	6.222 (0.2727–3.383)	
	Hyderabad	9	7	0.730	0.638	1.14386	0.253	2.074 (0.5205–1.980)	
	Islamabad	1	0	−16.585	3,956.180	−0.00419	0.997	6.27e-8 (−7770.5562–7737.386)	
	Karachi	2	1	0.288	1.286	0.22377	0.823	1.333 (−22321–2.807)	
	Karimabad	16	4	−0.405	0.682	−0.59443	0.552	0.667 (−1.7424–0.931)	
	Khudabad	4	2	0.288	0.950	0.30278	0.762	1.333 (−1.57465–2.150)	
	Khyber	8	3	−1.33e-14	0.782	−1.70e-14	1.000	1.000 (−1.5322–1.532)	
	Misgar	1	3	2.079	1.219	1.70577	0.088	8.000 (−0.3099–4.469)	
	Morkhun	0	2	18.547	2,797.442	0.00663	0.995	1.13e+8 (5464.3386–5501.432)	
	Murtazabad	10	8	0.758	0.615	1.23274	0.218	2.133 (−0.4470–1.962)	
	Nasirabad	1	1	0.981	1.467	0.66849	0.504	2.667 (−1.8949–3.857)	
	Nazimabad	0	1	18.547	3,956.180	0.00469	0.996	1.13e+8 (7735.4241–7772.518)	
	Passu	0	5	18.547	1,769.258	0.01048	0.992	1.13e+8 (−3449.1344–3486.228)	
	Shimshal	9	7	0.730	0.638	1.14386	0.253	2.074 (0.5205–1.980)	
	Shishkat	9	9	0.981	0.612	1.60169	0.109	2.667 (0.2194–2.181)	
	Sost	10	5	0.288	0.673	0.42753	0.669	1.333 (−1.0312–1.607)	
	Sultanabad	1	0	−16.585	3,956.180	−0.00419	0.997	6.27e-8 (−7770.55562–7737.386)	
Occupation	Farmer	32	28	−1.93	1.11	−1.7334	0.083	0.1458 (−4.102–0.2516)	0.0300
	Animal keeper (base)	1	6	NA	NA	NA	NA	NA	
	Butcher	7	4	−2.35	1.25	−1.8829	0.060	0.0952 (−4.799–0.0962)	
	Shepherd	13	5	−2.75	1.20	−2.2865	0.022^*^	0.0641 (−5.102– (−0.3924)	
	Business	20	22	−1.70	1.12	−1.5100	0.131	0.1833 (−3.898–0.5055)	
	Professional job	49	23	−2.55	1.11	−2.2970	0.022^*^	0.0782 (−4.722– (−0.3739)	
	Government service	29	31	−1.73	1.11	−1.5533	0.120	0.1782 (−3.902–0.456)	
	Other	52	40	−2.05	1.10	−1.8667	0.062	0.1282 (−4.211–0.1026)	
	Housewife	1	0	−15.36	535.41	−0.0287	0.977	2.14e-7 (−1064.747–1034.0309)	
	Student	14	10	−2.13	1.16	−1.8398	0.066	0.1190 (−4.395–0.1390)	
Religion	Muslim (base)	218	168	NA	NA	NA	NA	NA	
	Other	0	1	13.827	535.411	0.0258	0.979	1.01e+6 (0.000–inf)	
Ethnicity	Brusho (base)	169	125	NA	NA	NA	NA	NA	0.00213
	Wakhi	48	42	0.168	0.242	0.694	0.487	1.183 (−0.306–0.6423)	
	Not available	1	2	0.995	1.230	0.808	0.419	2.704 (−1.417–3.4063)	
Village	Aliabad (base)	22	13	NA	NA	NA	NA	NA	0.110
	Altit	10	10	0.5261	0.568	0.92657	0.354	1.692 (−0.587–1.639)	
	Attabad	0	2	18.0922	2,797.442	0.00647	0.995	7.20e+7 (−5464.793–5500.978)	
	Chipurson	0	6	18.0922	1,615.104	0.01120	0.991	7.20e+7 (−3147.453–3183.638)	
	Dorkhun	8	8	0.5261	0.610	0.86213	0.389	1.692 (−0.670–1.722)	
	Ganish	14	9	0.0843	0.552	0.15259	0.879	1.088 (−0.998–1.167)	
	Garelth	10	3	−0.6779	0.745	−0.90934	0.363	0.508 (−2.139–0.783)	
	Ghulkin	8	0	−17.0400	1,398.721	−0.01218	0.990	3.98e-8 (−2758.483–2724.403)	
	Gilgit	3	2	0.1206	0.978	0.12339	0.902	1.128 (−1.795–2.037)	
	Gulmit	9	5	−0.0617	0.658	−0.09370	0.925	0.940 (−1.352–1.229)	
	Hassanabad	12	2	−1.2657	0.840	−1.50663	0.132	0.282 (−2.912–0.381)	
	Hunza	13	10	0.2637	0.547	0.48206	0.630	1.302 (−0.809–1.336)	
	Hussaini	4	7	1.0857	0.718	1.51255	0.130	2.962 (−0.321–2.493)	
	Hyderabad	11	15	0.8362	0.529	1.58049	0.114	2.308 (−0.201–1.873)	
	Jutial	0	1	18.0922	3,956.180	0.00457	0.996	7.20e+7 (−7735.879–7772.063)	
	Karimabad	16	9	−0.0493	0.544	−0.09056	0.928	0.952 (−1.116–1.017)	
	Khanabad	2	2	0.5261	1.059	0.49658	0.619	1.692 (−1.550–2.603)	
	Khudabad	5	3	0.0153	0.810	0.01885	0.985	1.015 (−1.572–1.602)	
	Khyber	8	3	−0.4547	0.762	−0.59673	0.551	0.635 (−1.948–1.039)	
	Mayoon	1	0	−17.0400	3,956.180	−0.00431	0.997	3.98e-8 (−7771.011–7736.931)	
	Misgar	4	3	0.2384	0.840	0.28380	0.777	1.269 (−1.408–1.885)	
	Morkhun	0	2	18.0922	2,797.442	0.00647	0.995	7.20e+7 (−5464.793–5500.978)	
	Murtazabad	19	12	0.0666	0.508	0.13095	0.896	1.069 (−0.930–1.063)	
	Nasirabad	2	2	0.5261	1.059	0.49658	0.619	1.692 (−1.550–2.603)	
	Nazimabad	0	2	18.0922	2,797.442	0.00647	0.995	7.20e+7 (−5464.793–5500.978)	
	Nomal	0	2	18.0922	2,797.442	0.00647	0.995	7.20e+7 (−5464.793–5500.978)	
	Oshikhandass	0	1	18.0922	3,956.180	0.00457	0.996	7.20e+7 (−7735.879–7772.063)	
	Passu	1	4	1.9124	1.171	1.63245	0.103	6.769 (−0.384–4.208)	
	Rahimabad	1	1	0.5261	1.457	0.36112	0.718	1.692 (−2.329–3.381)	
	Shimshal	11	11	0.5261	0.552	0.95386	0.340	1.692 (−0.555–1.607)	
	Shishkat	13	14	0.6002	0.520	1.15353	0.249	1.822 (−0.420–1.620)	
	Sost	10	4	−0.3902	0.687	−0.56773	0.570	0.677 (−1.737–0.957)	
	Sultanabad	1	1	0.5261	1.457	0.36112	0.718	1.692 (−2329–3.381)	
Number of family members	2	0	1	NA	NA	NA	NA	NA	0.0631
	3	5	3	−17.1	2,400	−0.00712	0.994	3.83e0–8 (−4720–4686)	
	4	26	11	−17.4	2,400	−0.00726	0.994	2.70e0–8 (−4720–4686)	
	5	44	38	−16.7	2,400	−0.00696	0.994	5.52e0–8 (−4720–4686)	
	6	40	47	−16.4	2,400	−0.00684	0.995	7.51e0–8 (−4719–4687)	
	7	29	36	−16.3	2,400	−0.00681	0.995	7.93e0–8 (−4719–4687)	
	8	23	16	−16.9	2,400	−0.00706	0.994	4.44e0–8 (−4720–4686)	
	9	19	9	−17.3	2,400	−0.00722	0.994	3.03e0–8 (−4720–4686)	
	10	12	3	−18.0	2,400	−0.00748	0.994	1.60e0–8 (−4721–4685)	
	11	3	1	−17.7	2,400	−0.00736	0.994	2.13e0–8 (−4721–4685)	
	12	4	4	−16.6	2,400	−0.00690	0.994	6.39e0–8 (−4720–4686)	
	13	1	0	−33.1	3,393	−0.00976	0.992	4.08e-15 (−6684–6618)	
	14	3	0	−33.1	2,771	−0.01196	0.990	4.08e-15 (−5464–5397)	
	15	4	0	−33.1	2,683	−0.01235	0.990	4.08e-15 (−5291–5225)	
	16	2	0	−33.1	2,939	−0.01127	0.991	4.08e-15 (−5793–5727)	
	17	1	0	−33.1	3,393	−0.00976	0.992	4.08e-15 (−6684–6618)	
	18	2	0	−33.1	2,939	−0.01127	0.991	4.08e-15 (−5793–5727)	

* indicates the values which are significant.

**Table 10:** Association between landscape epidemiology and sociodemographic factors.

**Variables**	**Characteristic**	**Landscape epidemiology status**		**Estimate**	**SE**	***Z*-value**	***P*-value**	**Odd ratio**	***R* ^2^_McF_**
		**Good**	**Poor**						
Gender	Male	111	83	−0.00905	0.205	−0.0441	0.965	0.991 (−0.412–0.39356)	3.67e-6
	Female (Base)	110	83	NA	NA	NA	NA	NA	
Age	15–20 (Base)	27	18	NA	NA	NA	NA	NA	0.0190
	21–25	55	39	0.0617	0.369	0.1670	0.867	1.064 (−0.662–0.786)	
	26–30	23	17	0.1032	0.441	0.2337	0.815	1.109 (−0.762–0.968)	
	31–35	24	19	0.1719	0.432	0.3975	0.691	1.187 (−0.675–1.019)	
	36–40	31	22	0.0625	0.413	0.1515	0.880	1.065 (−0.746–0.871)	
	41–45	29	23	0.1737	0.413	0.4205	0.674	1.190 (−0.636–0.983)	
	46–50	5	9	0.9933	0.635	1.5632	0.118	2.700 (−0.252–2.239)	
	51–55	13	8	−0.0800	0.543	−0.1475	0.883	0.923 (−1.144–0.984)	
	56–60	2	3	0.8109	0.962	0.8427	0.399	2.250 (−1.075–2.697)	
	61–65	4	5	0.6286	0.737	0.8534	0.393	1.875 (−0.815–2.072)	
	71–75	5	0	−15.1606	650.874	−0.0233	0.981	2.61e-7 (−1290.849–1260.528)	
	76–80	2	2	0.4055	1.045	0.3879	0.698	1.500 (−1.643–2.454)	
	80+	1	1	0.4055	1.447	0.2803	0.779	1.500 (−2.430–3.241)	
Education	No formal education	50	43	0.8300	0.708	1.1719	0.241	2.293 (−0.558–2.218)	0.0238
	Below elementary (base)	8	3	NA	NA	NA	NA	NA	
	Elementary	12	3	−0.4055	0.935	−0.4335	0.665	0.667 (−2.239–1.428)	
	Secondary	19	13	0.6013	0.767	0.7843	0.433	1.825 (−0.901–2.104)	
	Higher secondary	28	33	1.1451	0.724	1.5814	0.114	3.143 (−0.274–2.564)	
	Undergraduate	65	52	0.7577	0.702	1.0792	0.281	2.133 (−0.618–2.134)	
	Graduate	8	7	0.8473	0.852	0.9943	0.320	2.333 (−0.823–2.518)	
	Post-graduate	31	12	0.0317	0.758	0.0419	0.967	1.032 (−1.453–1.517)	
Residency	Rural (base)	189	155	NA	NA	NA	NA	NA	0.0118
	Urban	32	11	−0.870	0.366	−2.38	0.017^*^	0.419 [−1.587– (−0.1523)]	
Marital status	Married (base)	122	97	NA	NA	NA	NA	NA	7.62e-4
	Unmarried	99	69	−0.132	0.208	−0.634	0.526	0.877 (−0.539–0.2752)	
Status	Employee (base)	82	60	NA	NA	NA	NA	NA	8.72e-4
	Student	52	44	0.1453	0.266	0.546	0.585	1.156 (−0.376–0.6669)	
	Other	87	62	−0.0264	0.238	−0.111	0.912	0.974 (−0.492–0.4394)	
Monthly income	<40	84	67	0.1231	0.229	0.5382	0.590	1.131 (−0.325–0.5712)	0.00121
	40–100 k (base)	95	67	NA	NA	NA	NA	NA	
	Above 100 k	13	12	0.2691	0.431	0.6245	0.532	1.309 (−0.575–1.1138)	
	No response	29	20	−0.0224	0.332	−0.0675	0.946	0.978 (−0.672–0.6275)	
Birthplace	Aliabad (base)	25	8	NA	NA	NA	NA	NA	0.0618
	Altit	9	6	0.7340	0.665	1.10302	0.270	2.083 (−0.5702–2.038)	
	Attabad	2	2	1.1394	1.079	1.05567	0.291	3.125 (−0.9761–3.255)	
	Chipurson	3	3	1.1394	0.912	1.24944	0.212	3.125 (−0.6480–2927)	
	Dorkhun	6	1	−0.6523	1.154	−0.56528	0.572	0.521 (−2.9141–1.609)	
	Ganish	10	10	1.1394	0.604	1.88601	0.059	3.125 (−0.0447–2.324)	
	Garelth	6	7	1.2936	0.689	1.87787	0.060	3.646 (−0.0565–2.644)	
	Ghulkin	1	6	2.9312	1.154	2.54008	0.011^*^	18.750 (0.6694–5.193)	
	Gilgit	11	10	1.0441	0.597	1.75018	0.080	2.841 (−0.1252–2213)	
	Gojal	1	0	−14.4266	1,455.398	−0.00991	0.992	5.43e-7 (−28866.9535–2838.100)	
	Gulmit	7	8	1.2730	0.658	1.93484	0.053	3.571 (−0.0165–2.562)	
	Hassanabad	7	7	1.1394	0.671	1.69722	0.090	3.125 (−0.1764–2.455)	
	Hunza	48	34	0.7946	0.464	1.71269	0.087	2.214 (−0.1147–1.704)	
	Hussaini	5	5	1.1394	0.752	1.51588	0.130	3.125 (−0.3338–2.613)	
	Hyderabad	8	8	1.1394	0.644	1.76875	0.077	3.125 (−0.1232–2.402)	
	Islamabad	0	1	16.7055	1,455.398	0.01148	0.991	1.80e+7 (−2835.8214–2869.232)	
	Karachi	2	1	0.4463	1.290	0.34587	0.729	1.563 (−2.0828–2.975)	
	Karimabad	15	5	0.0408	0.657	0.06213	0.950	1.042 (−1.2469–1.329)	
	Khudabad	3	3	1.1394	0.912	1.24944	0.212	3.125 (−0.6480–2.927)	
	Khyber	8	3	0.1586	0.790	0.20089	0.841	1.172 (−1.3888–1.707)	
	Misgar	3	1	0.0408	1.224	0.03335	0.973	1.042 (−2.3583–2.440)	
	Morkhun	2	0	−14.4266	1,029.122	−0.01402	0.989	5.43e-7 (−2031.4678–2002.615)	
	Murtazabad	9	9	1.1394	0.622	1.83109	0.067	3.125 (−0.0802–2.359)	
	Nasirabad	1	1	1.1394	1.471	0.77439	0.439	3.125 (−1.7444–4.023)	
	Nazimabad	1	0	−14.4266	1,455.398	−0.00991	0.992	5.43e-7 (−2866.9535–2838.100)	
	Passu	1	4	2.5257	1.190	2.12329	0.034	12.500 (0.1943–4.857)	
	Shimshal	8	8	1.1394	0.644	1.76875	0.077	3.125 (−0.1232–2.402)	
	Shishkat	11	7	0.6874	0.631	1.08863	0.276	1.989 (−0.5502–1.925)	
	Sost	8	7	1.0059	0.658	1.52892	0.126	2.734 (−0.2836–2.295)	
	Sultanabad	0	1	16.7055	1,455.398	0.01148	0.991	1.80e+7 (−2835.8214–2869.232)	
Occupation	Farmer	35	25	−0.0488	0.807	−0.0604	0.952	0.952 (−1.63–1.534)	0.0193
	Animal keeper	4	3	NA	NA	NA	NA	NA	
	Butcher	10	1	−2.0149	1.297	−1.5530	0.120	0.133 (−4.56–0.528)	
	Shepherd	8	10	0.5108	0.899	0.5682	0.570	1.667 (−1.25–2.273)	
	Business	24	18	−1.99e-14	0.825	−2.14e-14	1.000	1.000 (−1.62–1.617)	
	Professional job	44	28	−0.1643	0.801	−0.2051	0.837	0.848 (−1.73–1.406)	
	Government service	31	29	0.2210	0.806	0.2741	0.784	1.247 (−1.36–1.801)	
	Other	52	40	0.0253	0.792	0.0320	0.975	1.026 (−1.53–1.578)	
	Housewife	1	0	−13.2784	535.412	−0.0248	0.980	1.71e-6 (−1062.67–1036.109)	
	Student	12	12	0.2877	0.866	0.3322	0.740	1.333 (−1.411.985)	
Religion	Muslim (base)	221	165	NA	NA	NA	NA	NA	0.00321
	Other	0	1	13.858	535.411	0.0259	0.979	1.04e+6 (−1035.528–1063.2449)	
Ethnicity	Brusho (base)	170	124	NA	NA	NA	NA	NA	0.00746
	Wakhi	48	42	0.182	0.242	0.7518	0.452	1.200 (−0.292–0.6564)	
	Not available	3	0	−14.251	509.652	−0.0280	0.978	6.47e-7 (−1013.150–984.6493)	
Village	Aliabad (base)	22	13	NA	NA	NA	NA	NA	0.0664
	Altit	13	7	−0.0929	0.585	−0.15890	0.874	0.911 (−1.239–1.054)	
	Attabad	2	0	−16.0400	1,696.734	−0.00945	0.992	1.08e-7 (−3341.578–3309.498)	
	Chipurson	4	2	−0.1671	0.934	−0.17886	0.858	0.846 (−1.998–1.664)	
	Dorkhun	12	4	−0.5725	0.675	−0.84810	0.396	0.564 (−1.895–0.751)	
	Ganish	11	12	0.6131	0.545	1.12573	0.260	1.846 (−0.454–1.681)	
	Garelth	6	7	0.6802	0.657	1.03508	0.301	1.974 (−0.608–1.968)	
	Ghulkin	2	6	1.6247	0.888	1.82904	0.067	5.077 (−0.116–3.366)	
	Gilgit	3	2	0.1206	0.978	0.12339	0.902	1.128 (−1.795–2.037)	
	Gulmit	6	8	0.8138	0.643	1.26468	0.206	2.256 (−0.447–2.075)	
	Hassanabad	7	7	0.5261	0.639	0.82354	0.410	1.692 (−0.726–1.778)	
	Hunza	15	8	−0.1025	0.560	−0.18293	0.855	0.903 (−1.201–0.996)	
	Hussaini	6	5	0.3438	0.699	0.49158	0.623	1.410 (−1.027–1.714)	
	Hyderabad	15	11	0.2159	0.529	0.40812	0.683	1.241 (−0.821–1.253)	
	Jutial	1	0	−16.0400	2,399.545	−0.00668	0.995	1.08e-7 (−4719.061–4686.981)	
	Karimabad	16	9	−0.0493	0.544	−0.09056	0.928	0.952 (−1.116–1.017)	
	Khanabad	2	2	0.5261	1.059	0.49658	0.619	1.692 (−1.550–2.603)	
	Khudabad	4	4	0.5261	0.789	0.66686	0.505	1.692 (−1.020–2.072)	
	Khyber	8	3	−0.4547	0.762	−0.59673	0.551	0.635 (−1.948–1.039)	
	Mayoon	0	1	17.0922	2,399.545	0.00712	0.994	2.65e+7 (−4685.929–4720.113)	
	Misgar	5	2	−0.3902	0.907	−0.43028	0.667	0.677 (−2168–1.387)	
	Morkhun	2	0	−16.0400	1,696.734	−0.00945	0.992	1.08e-7 (−3341.578–3309.498)	
	Murtazabad	17	14	0.3319	0.503	0.66041	0.509	1.394 (−0.653–1.317)	
	Nasirabad	3	1	−0.5725	1.207	−0.47452	0.635	0.564 (−2.937–1.792)	
	Nazimabad	2	0	−16.0400	1,696.734	−0.00945	0.992	1.08e-7 (−3341.578–3309.498)	
	Nomal	1	1	0.5261	1.457	0.36112	0.718	1.692 (−2329–3.381)	
	Oshikhandass	1	0	−16.0400	2,399.545	−0.00668	0.995	1.08e-7 (−4719.051–4686.981)	
	Passu	1	4	1.9124	1.171	1.63245	0.103	6.769 (−0.384–4.208)	
	Rahimabad	0	2	17.0922	1,696.734	0.01007	0.992	2.65e+7 (−3308.446–3342.630)	
	Shimshal	10	12	0.7084	0.553	1.28124	0.200	2.031 (−0.375–1.792)	
	Shishkat	16	11	0.1514	0.525	0.28830	0.773	1.163 (−0.878–1.181)	
	Sost	8	6	0.2384	0.643	0.37051	0.711	1.269 (−1.023–1.500)	
	Sultanabad	0	2	17.0922	1,696.734	0.01007	0.992	2.65e+7 (−3308.446–3342.630)	
Number of family members	2 (Base)	0	1	NA	NA	NA	NA	NA	0.0305
	3	3	5	−14.1	883	−0.0159	0.987	7.87e0–7 (−1744–1716)	
	4	18	19	−14.5	883	−0.0164	0.987	4.98e0–7 (−1745–1716)	
	5	52	30	−15.1	883	−0.0171	0.986	2.72e0–7 (−1745–1715)	
	6	48	39	−14.8	883	−0.0167	0.987	3.84e0–7 (−1745–1715)	
	7	43	22	−15.2	883	−0.0173	0.986	2.42e0–7 (−1745–1715)	
	8	20	19	−14.6	883	−0.0166	0.987	4.48e0–7 (−1745–1716)	
	9	13	15	−14.4	883	−0.0163	0.987	5.45e0–7 (−1745–1716)	
	10	11	4	−15.6	883	−0.0176	0.986	1.72e0–7 (−1746–1715)	
	11	2	2	−14.6	883	−0.0165	0.987	4.72e0–7 (−1745–1716)	
	12	3	5	−14.1	883	−0.0159	0.987	7.87e0–7 (−1744–1716)	
	13	0	1	5.72e-9	1,248	4.58e-12	1.000	1.00 (−2447–2447)	
	14	2	1	−15.3	883	−0.0173	0.986	2.36e0–7 (−1745–1715)	
	15	3	1	−15.7	883	−0.0177	0.986	1.57e0–7 (−1746–1714)	
	16	1	1	−14.6	883	−0.0165	0.987	4.72e0–7 (−1745–1716)	
	17	1	0	−29.1	1,248	−0.0233	0.981	2.23e-13 (−2476–2418)	

* indicates the values which are significant.

**Table 11:** Association between disease management and sociodemographic factors.

**Variables**	**Characteristic**	**Disease management status**		**Estimate**	**SE**	***Z*-value**	***P*-value**	**Odd ratio**	***R* ^2^_McF_**
		**Good**	**Poor**						
Gender	Male	133	61	0.192	0.223	0.859	0.390	1.212 (−0.246–0.630)	0.00157
	Female (Base)	140	53	NA	NA	NA	NA	NA	
Age	15–20 (Base)	37	8	NA	NA	NA	NA	NA	0.0386
	21–25	56	38	1.144	0.443	2.5821	0.010^*^	3.138 (0.27556–2.012)	
	26–30	25	15	1.021	0.509	2.0067	0.045^*^	2.775 (0.02378–2.018)	
	31–35	33	10	0.338	0.531	0.6353	0.525	1.402 (−0.70387–1.379)	
	36–40	41	12	0.303	0.510	0.5941	0.552	1.354 (−0.69610–1.302)	
	41–45	41	11	0.216	0.517	0.4174	0.676	1.241 (−0.79757–1.229)	
	46–50	10	4	0.615	0.709	0.8682	0.385	1.850 (−0.77352–2.004)	
	51–55	15	6	0.615	0.621	0.9910	0.322	1.850 (−0.60151–1.832)	
	56–60	2	3	1.937	0.993	1.9513	0.051	6.938 (−0.00862–3.883)	
	61–65	5	4	1.308	0.776	1.6862	0.092	3.700 (−0.21241–2.829)	
	71–75	3	2	1.126	0.993	1.1343	0.257	3.083 (−0.81955–3.072)	
	76–80	3	1	0.433	1.219	0.3552	0.722	1.542 (−1.95585–2.822)	
	80+	2	0	−13.035	624.194	−0.0209	0.983	2.18e-6 (−1236.432–1210.363)	
Education	No formal education	74	19	−0.3788	0.724	−0.5230	0.601	0.685 (−1.798–1.041)	0.0155
	Below elementary (base)	8	3	NA	NA	NA	NA	NA	
	Elementary	11	4	−0.0308	0.894	−0.0344	0.973	0.970 (−1.783–1.721)	
	Secondary	20	12	0.4700	0.769	0.6110	0.541	1.600 (−1.038–1.978)	
	Higher secondary	43	18	0.1100	0.733	0.1501	0.881	1.116 (−1.326–1.546)	
	Undergraduate	80	37	0.2097	0.706	0.2972	0.766	1.233 (−1.173–1.593)	
	Graduate	11	4	−0.0308	0.894	−0.0344	0.973	0.970 (−1.783–1.721)	
	Post-graduate	26	17	0.5559	0.745	0.7458	0.456	1.744 (−0.905–2.017)	
Residency	Rural (base)	240	104	NA	NA	NA	NA	NA	0.00199
	Urban	33	10	−0.358	0.380	−0.942	0.346	0.699 (−1.10–0.386)	
Marital status	Married (base)	167	52	NA	NA	NA	NA	NA	0.0168
	Unmarried	106	62	0.630	0.225	2.80	0.005	1.878 (0.189–1.072)	
Status	Employee (base)	99	43	NA	NA	NA	NA	NA	0.00487
	Student	63	33	0.187	0.282	0.664	0.507	1.206 (−0.365–0.740)	
	Other	111	38	−0.238	0.262	−0.908	0.364	0.788 (−0.752–0.276)	
Monthly income	<40	112	39	−0.277	0.251	−1.103	0.270	0.758 (−0.770–0.215)	0.00938
	40–100 k (base)	111	51	NA	NA	NA	NA	NA	
	Above 100 k	20	5	−0.609	0.528	−1.153	0.249	0.544 (−1.643–0.426)	
	No response	30	19	0.321	0.338	0.948	0.343	1.378 (−0.343–0.984)	
Birthplace	Aliabad (base)	27	6	NA	NA	NA	NA	NA	0.173
	Altit	7	8	1.6376	0.687	2.38474	0.017^*^	5.143 (0.2917–2984)	
	Attabad	4	0	−16.0620	1978.090	−0.00812	0.994	1.06e-7 (−3893.0476–3860.924)	
	Chipurson	3	3	1.5041	0.933	1.61220	0.107	4.500 (−0.3244–3.333)	
	Dorkhun	4	3	1.2164	0.887	1.37113	0.170	3.375 (−0.5224–2.955)	
	Ganish	13	7	0.8850	0.651	1.36001	0.174	2.423 (−0.3904–2.160)	
	Garelth	13	0	−16.0620	1097.247	−0.01464	0.988	1.06e-7 (−2166.6268–2134.503)	
	Ghulkin	6	1	−0.2877	1.171	−0.24575	0.806	0.750 (−2.5821–2.007)	
	Gilgit	17	4	0.0572	0.716	0.07984	0.936	1.059 (−1.3460–1.450)	
	Gojal	0	1	19.0701	3956.180	0.00482	0.996	1.91e+8 (−7734.9009–7773.041)	
	Gulmit	13	2	−0.3677	0.884	−0.41620	0.677	0.692 (−20994–1.364)	
	Hassanabad	12	2	−0.2877	0.887	−0.32428	0.746	0.750 (−2.0265–1.451)	
	Hunza	48	34	1.1592	0.504	2.30038	0.021^*^	3.187 (0.1715–2.147)	
	Hussaini	5	5	1.5041	0.777	1.93579	0.053	4.500 (−0.0188–3.027)	
	Hyderabad	15	1	−1.2040	1.127	−1.06820	0.285	0.300 (−3.4131–1.005)	
	Islamabad	1	0	−16.0620	3956.180	−0.00406	0.997	1.06e-7– (7770.0330–7737.909)	
	Karachi	1	2	2.1972	1.305	1.68336	0.092	9.000 (−0.3610–4.755)	
	Karimabad	8	12	1.9095	0.642	2.97482	0.003^*^	6.750 (0.6514–3.168)	
	Khudabad	2	4	2.1972	0.977	2.24992	0.024^*^	9.000 (0.2832–4.111)	
	Khyber	9	2	−6.05e-15	0.903	−6.70e-15	1.000	1.000 (−1.7692–1.769)	
	Misgar	1	3	2.6027	1.240	2.09933	0.036^*^	13.500 (0.1728–5.033)	
	Morkhun	1	1	1.5041	1.484	1.01320	0.311	4.500 (−1.4055–4414)	
	Murtazabad	12	6	0.8109	0.674	1.20392	0.229	2.250 (−0.5093–2.131)	
	Nasirabad	2	0	−16.0620	2,797.442	−0.00574	0.995	1.06e-7 (−5498.9475–5466.824)	
	Nazimabad	1	0	−16.0620	3,956.180	−0.00406	0.997	1.06e-7 (−7770.0330–7737.909)	
	Passu	5	0	−16.0620	1,769.258	−0.00908	0.993	1.06e-7 (−3483.7433–3451.619)	
	Shimshal	14	2	−0.4418	0.880	−0.50185	0.616	0.643 (−21674–1.284)	
	Shishkat	18	0	−16.0620	932.481	−0.01723	0.986	1.06e-7 (−1843.6907–1811.567)	
	Sost	10	5	0.8109	0.710	1.14260	0.253	2.250 (−0.5801–2.202)	
	Sultanabad	1	0	−16.0620	3,956.180	−0.00406	0.997	1.06e-7 (−7770.0330–7737.909)	
Occupation	Farmer	45	15	−0.1823	0.888	−0.2053	0.837	0.833 (−1.92–1.559)	0.0145
	Animal keeper (base)	5	2	NA	NA	NA	NA	NA	
	Butcher	7	4	0.3567	1.045	0.3412	0.733	1.429 (−1.69–2.406)	
	Shepherd	13	5	−0.0392	0.988	−0.0397	0.968	0.962 (−1.98–1.898)	
	Business	31	11	−0.1198	0.907	−0.1320	0.895	0.887 (−1.90–1.658)	
	Professional job	50	22	0.0953	0.875	0.1089	0.913	1.100 (−1.621.810)	
	Government service	47	13	−0.3689	0.893	−0.4129	0.680	0.691 (−2.12–1.382)	
	Other	57	35	0.4286	0.864	0.4962	0.620	1.535 (−1.26–2.122)	
	Housewife	1	0	−12.6498	535.412	−0.0236	0.981	3.21e-6 (−1062–04–1036.738)	
	Student	17	7	0.0290	0.950	0.0305	0.976	1.029 (−1.83–1.890)	
Religion	Muslim (base)	273	113	NA	NA	NA	NA	NA	0.00522
	Other	0	1	14.448	535.411	0.0270	0.978	1.88e+6 (−1034.835–1063.835)	
Ethnicity	Brusho (base)	208	86	NA	NA	NA	NA	NA	0.00472
	Wakhi	62	28	0.0883	0.261	0.3378	0.736	1.092 (−0.424–0.600)	
	Not available	3	0	−14.6829	840.274	−0.0175	0.986	4.20e-7 (−1661.590–1632.224)	
Village	Aliabad (base)	20	15	NA	NA	NA	NA	NA	0.191
	Altit	11	9	0.0870	0.565	0.15413	0.878	1.0909 (−1.019–1.1935)	
	Attabad	2	0	−17.2784	2,797.442	−0.00618	0.995	3.13e-8 (−5500.164–5465.6071)	
	Chipurson	3	3	0.2877	0.885	0.32504	0.745	1.3333 (−1.447–2.0224)	
	Dorkhun	11	5	−0.5008	0.638	−0.78440	0.433	0.6061 (−1.752–0.7505)	
	Ganish	16	7	−0.5390	0.567	−0.94982	0.342	0.5833 (−1.651–0.5732)	
	Garelth	13	0	−17.2784	1,097.247	−0.01575	0.987	3.13e-8 (−2167.843–2133.2863)	
	Ghulkin	6	2	−0.8109	0.885	−0.91624	0.360	0.4444 (−2546–0.9238)	
	Gilgit	5	0	−17.2784	1,769.258	−0.00977	0.992	3.13e-8 (−3484.950–3450.4029)	
	Gulmit	13	1	−2.2773	1.093	−2.08443	0.037^*^	0.1026 [−4.419– (−0.1360)]	
	Hassanabad	13	1	−2.2773	1.093	−2.08443	0.037^*^	0.1026 [−4.419– (−0.1360)]	
	Hunza	18	5	−0.9933	0.610	−1.62801	0.104	0.3704 (−2.89–0.2025)	
	Hussaini	5	6	0.4700	0.695	0.67605	0.499	1.6000 (−0.893–1.8326)	
	Hyderabad	21	5	−1.1474	0.604	−1.90106	0.057	0.3175 (−2.330–0.0356)	
	Jutial	1	0	−17.2784	3,956.180	−0.00437	0.997	3.13e-8 (−7771.249–7736.6826)	
	Karimabad	12	13	0.3677	0.526	0.69878	0.485	1.4444 (−0.664–1.3991)	
	Khanabad	0	4	17.8538	1,978.090	0.00903	0.993	5.67e+7 (−3859.132–3894.8393)	
	Khudabad	3	5	0.7985	0.806	0.99043	0.322	2.2222 (−0.782–2.3787)	
	Khyber	9	2	−1.2164	0.853	−1.42585	0.154	0.2963 (−2.888–0.4556)	
	Mayoon	1	0	−17.2784	3,956.180	−0.00437	0.997	3.13e-8 (−7771.249–7736.6926)	
	Misgar	2	5	1.2040	0.904	1.33228	0.183	3.3333 (−0.567–2.9752)	
	Morkhun	1	1	0.2877	1.455	0.19774	0.843	1.3333 (−2.564–3.1392)	
	Murtazabad	19	12	−0.1719	0.503	−0.34191	0.732	0.8421 (−1.157–0.8133)	
	Nasirabad	4	0	−17.2784	1,978.090	−0.00873	0.993	3.13e-8 (−3894.254–3859.7072)	
	Nazimabad	2	0	−17.2784	2,797.442	−0.00618	0.995	3.13e-8 (−5500.164–5465.6071)	
	Nomal	2	0	−17.2784	2,797.442	−0.00618	0.995	3.13e-8 (−5500.164–5465.6071)	
	Oshikhandass	1	0	−17.2784	3,956.180	−0.00437	0.997	3.13e-8 (−7771.249–7736.6926)	
	Passu	5	0	−17.2784	1,769.258	−0.00977	0.992	3.13e-8 (−3484.960–3450.4029)	
	Rahimabad	0	2	17.8538	2,797.442	0.00638	0.995	5.67e+7 (−5465.032–5500.7392)	
	Shimshal	17	5	−0.9361	0.613	−1.52764	0.127	0.3922 (−2.137–0.2649)	
	Shishkat	26	1	−2.9704	1.075	−2.76377	0.006^*^	0.0513 [−5.077– (−0.8639)]	
	Sost	10	4	−0.6286	0.683	−0.92019	0.357	0.5333 (−1.958–0.7103)	
	Sultanabad	1	1	0.2877	1.455	0.19774	0.843	1.3333 (−2.564–3.1392)	
Number of family members	2 (Base)	1	0	NA	NA	NA	NA	NA	0.0330
	3	4	4	15.6	1,455	0.01070	0.991	5.76e+6 (−2837–2868)	
	4	28	9	14.4	1,455	0.00992	0.992	1.85e+6 (−2838–2867)	
	5	59	23	14.6	1,455	0.01005	0.992	2.24e+6 (−2837–2868)	
	6	66	21	14.4	1,455	0.00991	0.992	1.83e+6 (−2837–2868)	
	7	45	20	14.8	1,455	0.01014	0.992	2.56e+6 (−2837–2868)	
	8	26	13	14.9	1,455	0.01022	0.992	2.88e+6 (−2837–2868)	
	9	19	9	14.8	1,455	0.01018	0.992	2.73e+6 (−2837–2868)	
	10	11	4	14.6	1,455	0.01000	0.992	2.09e+6 (−2837–2868)	
	11	2	2	15.6	1,455	0.01070	0.991	5.76e+6 (−2837–2868)	
	12	2	6	16.7	1,455	0.01145	0.991	1.73e+7 (−2836–2869)	
	13	1	0	−3.64e-8	2,058	−1.77e-11	1.000	1.000 (−4034–4034)	
	14	2	1	14.9	1,455	0.01022	0.992	2.88e+6 (−2838–2867)	
	15	3	1	14.5	1,455	0.00994	0.992	1.92e+6 (−2838–2867)	
	16	1	1	15.6	1,455	0.01070	0.991	5.76e+6 (−2837–2868)	
	17	1	0	−3.64e-8	2,058	−1.77e-11	1.000	1.000 (−4034–4034)	
	18	2	0	−3.64e-8	1,782	−2.04e-11	1.000	1.000 (−3494–3494)	

* indicates the values which are significant.

## Discussion

AE is a zoonotic parasitic disease caused by *E. multilocularis* in humans and rodents. AE substantially endangers people's health and safety, and has an impact on social and economic development (17). Pakistan is a developing country of low socio-economic standing. The large majority of Pakistanis are involved in agriculture and small-scale dairy farming. Workers on these often small farms have direct contact with animals and, because health and hygiene principles are not adequately observed, residents of these places are at high risk of AE (18).

## Sociodemographic factors

Age contributed to the overall models that determined knowledge and perceptions of AE. Elevated contaminations in the age range 20–59 years is most likely attributable to occupation and close interaction with canines and the environment (19). A comparable study in Jordan found that children had the highest risk of infection. Furthermore, Kyrgyzstan has its highest prevalence among children aged 16 years (20). According to a study conducted in 2004, farmers were at a significant risk of infection due to the exposure and contact with contaminated objects, which is consistent with other research that found that farming was responsible for 65% of cases (21). The other risk factors posed infection includes forestry, hunting, and simply living in a disease cluster or rural location (21, 22). According to a survey performed in east Gansu, China, females had a considerably higher prevalence of AE than males, which was consistent with findings in other endemic locations (23).

According to a study conducted in China's Tibet Autonomous Region, illiterate middle-aged and older women were more likely to have AE than the general population. Middle-aged and older women are designated vulnerable populations as they are more likely to come into contact with diseased pets and contaminated environments. Women in this community are more likely to perform housework, which includes tasks such as collecting and burning yak dung (the primary fuel in pastoral areas) and feeding dogs. In addition, young and middle-aged males in vulnerable communities may be predisposed to echinococcosis because of inappropriate behavior especially regarding poor habit of handwashing, poor knowledge and occupations like herdsmen and farmer which increase the risk of exposure to *Echinococcus* eggs (23, 24).

Sociodemographic factors such as gender, age, marital status, birthplace, occupation, residency, religion and income are important in determining the association of disease with KAPs.

## Knowledge about AE

Our results indicated that knowledge about AE was poor in Hunza. The majority of the 387 respondents had never heard of any parasitic diseases. Knowledge about the cycle of transmission and infection was low among the participants, who lacked knowledge about the disease hosts. Knowledge about AE is important to effective prevention and control strategies and the participants were evidently unaware of the factors responsible for the prevalence and development of infection in either humans or animals.

Echinococcosis transmission has been demonstrated as strongly related to educational level, human behavior, lifestyle, living habits, and environmental factors (17). Health education plays a critical role in lowering echinococcosis transmission to humans (25). To prevent and control echinococcosis, raising awareness about lifestyle and living habits that increase susceptibility to echinococcosis in the general population is necessary. Residents need guidance to develop positive attitudes toward echinococcosis prevention and control, as well as to modify habits that increase risk of infection. Notably, echinococcosis is frequently endemic in underdeveloped or resource-poor regions where education is weak and illiteracy is rampant (26, 27).

A study from China likewise indicated that education level influenced views and practices regarding AE. Study data revealed that as education levels increased, the risk of echinococcosis is decreased. The study also found that just a tiny percentage of the interviewed locals were well versed in all aspects of echinococcosis. Many people were unaware of the routes of echinococcosis infection in humans or canines. The paucity of knowledge about echinococcosis infection routes resulted in a lack of knowledge about disease prevention. Changes in practice are heavily influenced by knowledge and attitudes. However, there is need of awareness to improve echinococcosis-related knowledge and change population attitudes in Pakistan (17, 23).

### Attitudes toward AE

Perception about a disease can influence its epidemiology. If people perceive themselves at risk of acquiring the disease, they are more likely to protect themselves from infection with the disease and vice versa. The majority of respondents in our study did not believe themselves at risk of infection with *E. multilocularis*. However, they expressed a positive attitude toward preventing infection.

A study by Ahmed (28) that explored the relationship between dog populations and prevalence of echinococcal disease found that humans who come into contact with dogs are at least twice as likely to acquire an echinococcal infection than those who do not have contact with dogs. This is because infection is more likely in a polluted environment containing infected dog feces, which contain parasite eggs. In the same study, Khartoum city had the highest rate of echinococcosis seropositivity among humans (14.8%), most likely owing to the presence of a large dog population in the area. According to another study, AE is common among hunters, trappers, and others who work with fox fur. Hyperendemic foci have been seen in certain Inuit towns in the North American tundra, and in western China, where native dogs eat infected commensal rodents daily (29).

Another study reported that the Qinghai-Tibet Plateau has a vast landscape with diverse populations of wild canids, rats, and other wild animals, and, because the majority of the local people are herders, the natural AE transmission cycle is widespread. This region has many domestic and stray dogs, which can be considered a major source of infection as terminal hosts (30). These ecological factors could be key determinants of AE (31). Educating local people and primary-level health workers about AE will assist in promoting practices that prevent AE in humans.

### Practices associated with AE

Several practices were associated with AE in our study. In the surveyed areas where stray dogs and wild canids were present thus risk of developing AE might be high. Of the total participants, 10.8% owned dogs. Most of the participants indicated the presence of rodents on grazing land. Rodents are intermediate hosts of *E. multilocularis* and stray and wild animals prey on these rodents, initiating the cycle of infection. Source drinking water was from glaciers. Participants indicated positively that they practiced self-hygiene such as washing hands, using gloves while handling soil and washing vegetables before eating.

Tibetan people keep large numbers of dogs, which are mostly employed to guard property and animals. According to one of previous research, 82.4% of the population of Tibet had dogs, with 21% owning three or more. Because it is against Buddhist doctrine to kill animals, including dogs, vast numbers of stray dogs cluster around temples and townships, where they are fed by monks and herders. Dogs are also predators of small mammals on nearby pastures. A necropsy of the intestines of stray dogs in this location revealed a prevalence of *E. granulosus* of 29.5% and *E. multilocularis* of 11.5% (31, 32).

Because of their frequent interaction with humans and contamination of soil around residences and in gardens, pet animals may constitute a risk for AE. We reported a link between dog ownership and AE, as well as a weaker but still significant link between having cats that wander freely outside or consume mice as shown in association table. “Dogs that killed game,” a rare action by individual dogs, was the factor in our survey that had the strongest link to the disease. As a result, the attributable risk was lower than for the other dog-related variables. Several other studies have found that dogs and cats are significant risk factors for AE, although results have been mixed (33, 34). The number of dogs owned over time and the degree of dog contact were the most relevant risk factors in a Chinese study of the Gansu-Han population with over 2,500 participants, including 86 individuals with AE (12).

The only two food consumption factors linked to AE were chewing grass and consuming unclean strawberries, in one study. Ingestion of eggs from infected plant parts or soil-stained hands also increased risk. Other garden food, as well as mushrooms from fields and meadows, were only eaten uncooked and unwashed on rare occasions (33).

### Landscape epidemiology of AE

According to recent estimates, AE is subject to global spread and is an emerging and re-emerging problem in many areas. AE endemicity varies geographically, and it may be influenced by global environmental change over time. Landscape epidemiology provides a unique opportunity to assess and predict infection risk in several spatial and temporal dimensions (34). Environmental factors that support fluctuation in host population densities are likely to influence the risk of human infection (34, 35).

The participants in our study had poor knowledge of landscape epidemiology and its impact on the prevalence of infection in the area. They had poor knowledge of the environmental factors that affect AE prevalence and how environmental factors influence the level of transmission of infection and disease risk depending on critical habitat and spatial contribution. The majority of the participants were unaware of AE's dependency on the connectivity of vector, host and human behavior as an important controlling factor of vector-human contact and infection.

With respect to the transmission ecology of *E. multilocularis* and the epidemiology of human AE, the function of the physical environment, particularly landscape characteristics, is becoming increasingly important. In China, landscape ecology methodologies for assessing human AE risk have been examined (12, 17, 36). A study conducted to understand landscape epidemiology in southern Ningxia, China reported that in Xiji, possible *E. multilocularis* intermediate hosts could be found in every habitat. High densities of preferred susceptible prey provide ideal conditions for *E. multilocularis* transmission (36). Understanding the landscape epidemiology aspects of AE may also provide scientific information to enhance environmental policymaking and landscape planning procedures in echinococcosis-endemic locations. As a result, landscape epidemiology may be effective in promoting environmentally based interventions with minimum influence on *Echinococcus spp*. transmission dynamics. This is especially important in areas where climate change and landscape changes may be encouraging parasite transmission (35).

### Disease management of AE

Human AE control and prevention methods can be implemented at many levels. Individually, hygiene-related measures and periodic deworming of domestic dogs are essential methods for reducing exposure to infective parasite eggs. On an environmental level, efforts to limit contamination with infective *E. multilocularis* eggs are aimed at either direct parasite management through deworming definitive hosts or wildlife host population suppression. In the past, population control tactics for the fox as a definitive host, such as hunting, trapping, and culling, have been advocated. When considering interventions in host populations, however, ecological changes and their impact on host populations should be considered (24). By deworming red foxes based on regular baiting campaigns, Japan and France have demonstrated the feasibility of lowering the infection pressure with *E. multilocularis* eggs.

In our study, the majority of respondents had a positive attitude toward receiving screening, free treatment and, if needed, surgery for the disease. Respondents had good knowledge about the importance of vaccination campaigns for humans and animals and the deworming of dogs. The population overall gave a positive response and indicated good knowledge about the importance of awareness and proper treatment in prevention of AE.

A previous study reported that different risk factors plays an important role toward the transmission of AE. The treatment of foxes with praziquantel-impregnated baits can disrupt the wildlife cycle (19). However, the feasibility of implementing such controls throughout wide swathes of AE-prevalent areas, such as the Tibetan plateau, is debatable. Although dog interaction has been identified as a risk factor for transmission to humans (12) and dogs are particularly vulnerable to infection with this parasite (37), periodic treatment of dogs will not disrupt the transmission cycle and will have considerably less impact on long-term transmission rates to humans (38, 39). Other studies suggest that improved control of food or water supplies that may be contaminated with parasite eggs is another way to reduce disease burden. The attributable percentage of disease burden owing to these transmission pathways, as well as the cost-efficacy of such intervention techniques, would determine such control (19). If populations in which the prevalence of AE is increasing take preventive measures such as self-hygiene, proper inspection and treatment for disease, food inspection and deworming of the wild animals around them, the prevalence of disease and transmission of infection from animals to humans may be more adequately controlled.

## Conclusion

Our study indicated that awareness of AE was generally very low in the Hunza population of Pakistan. We found that the residents of Hunza did not know about AE, the threats associated with it, the life cycle of parasite *E. multilocularis*, its control or disease management. Many practices and factors were observed that could predispose the population to infection by AE. Wild animals were common in most villages. These factors favor the exposure of the population to *E. multilocularis*.

Despite low awareness of AE, positive attitudes toward the treatment of the disease were observed and the study population responded positively to questions that were important to controlling the disease. Interrupting the parasite life cycle and establishing and maintaining a proper surveillance system is crucial for the prevention and control of this disease. Creating awareness among residents of Hunza and other parts of Pakistan where the same risk factors are observed is necessary. As this disease is becoming more prevalent in Pakistan, improved awareness among the population will prevent future health problems. A proper surveillance system and further research on this zoonotic disease in humans are recommended.

## Data availability statement

The original contributions presented in the study are included in the article/supplementary material, further inquiries can be directed to the corresponding authors.

## Ethics statement

The studies involving human participants were reviewed and approved by Ethics Committee of COMSATS University Islamabad (CUI), Islamabad under no. ERB/18/72. Written informed consent to participate in this study was provided by the participants' legal guardian/next of kin.

## Author contributions

NJ collected the data and authored the paper following discussions with HA and JC. HA designed the study methodology. MSA, NJ, MA, SS, and JC helped in statistical analysis. MSA, SS, NJ, MA, CY, and JC revised the paper. All authors contributed to the article and approved the submitted version.

## Funding

This study was supported by the National Natural Science Foundation of China (Nos. 81971969 and 81772225 to JC) and the Three-Year Public Health Action Plan (2020–2022) of Shanghai (No. GWV-10.1-XK13 to JC). The funders had no role in the study design, the data collection and analysis, the decision to publish, or the preparation of the manuscript.

## Conflict of interest

The authors declare that the research was conducted in the absence of any commercial or financial relationships that could be construed as a potential conflict of interest.

## Publisher's note

All claims expressed in this article are solely those of the authors and do not necessarily represent those of their affiliated organizations, or those of the publisher, the editors and the reviewers. Any product that may be evaluated in this article, or claim that may be made by its manufacturer, is not guaranteed or endorsed by the publisher.

## Abbreviations

AE: Alveolar echinococcosis
WHO: World Health Organization
P.R. China: People's Republic of China.
